# Construction of m6A-based prognosis signature and prediction for immune and anti-angiogenic response

**DOI:** 10.3389/fmolb.2022.1034928

**Published:** 2022-10-21

**Authors:** Xiang-Xu Wang, Li-Hong Wu, Qiong-Yi Dou, Liping Ai, Yajie Lu, Shi-Zhou Deng, Qing-Qing Liu, Hongchen Ji, Hong-Mei Zhang

**Affiliations:** ^1^ Department of Clinical Oncology, Xijing Hospital, Fourth Military Medical University, Xi’an, Shaanxi, China; ^2^ Xijing 986 Hospital Department, Fourth Military Medical University, Xi’an, Shaanxi, China

**Keywords:** hepatocellular carcinoma, m6A modification, tumor microenvironment, prognostic signature, immunotherapy

## Abstract

**Background:** Increasing evidence illustrated that m6A regulator-mediated modification plays a crucial role in regulating tumor immune and angiogenesis microenvironment. And the combination of immune checkpoint inhibitor and anti-angiogenic therapy has been approved as new first-line therapy for advanced HCC. This study constructed a novel prognosis signature base on m6A-mediated modification and explored the related mechanism in predicting immune and anti-angiogenic responses.

**Methods:** Gene expression profiles and clinical information were collected from TCGA and GEO. The ssGSEA, MCPCOUNT, and TIMER 2.0 algorithm was used to Estimation of immune cell infiltration. The IC_50_ of anti-angiogenic drugs in GDSC was calculated by the “pRRophetic” package. IMvigor210 cohort and Liu et al. cohort were used to validate the capability of immunotherapy response. Hepatocellular carcinoma single immune cells sequencing datasets GSE140228 were collected to present the expression landscapes of 5 hub genes in different sites and immune cell subpopulations of HCC patients.

**Results:** Three m6A clusters with distinct immune and angiogenesis microenvironments were identified by consistent cluster analysis based on the expression of m6A regulators. We further constructed a 5-gene prognosis signature (termed as m6Asig-Score) which could predict both immune and anti-angiogenic responses. We illustrated that high m6Asig-Score is associated with poor prognosis, advanced TNM stage, and high TP53 mutation frequency. Besides, the m6Asig-Score was negatively associated with immune checkpoint inhibitors and anti-angiogenic drug response. We further found that two of the five m6Asig-Score inner genes, B2M and SMOX, were associated with immune cell infiltration, immune response, and the sensitivity to sorafenib, which were validated in two independent immunotherapy cohorts and the Genomics of Drug Sensitivity in Cancer (GDSC) database.

**Conclusion:** We constructed a novel prognosis signature and identified B2M and SMOX for predicting immune and anti-angiogenic efficacy in HCC, which may guide the combined treatment strategies of immunotherapy and anti-angiogenic therapy in HCC.

## Introduction

Hepatocellular carcinoma (HCC) is one of the most common malignant tumors and the third cause of cancer-related deaths in the world (Sung et al., 2021). Unfortunately, most patients with liver cancer are diagnosed at an advanced stage, and they have no chance of radical surgical resection (Xu et al., 2018). The combination treatment for advanced HCC clinical trials, IMbrave150 phase 3 trial (Atezolizumab plus Bevacizumab) (Finn et al., 2020a), ORIENT-32 phase 3 trial (Sintilimab plus bevacizumab similar IBI305) (Ren et al., 2021), COSMIC-312 (Cabozantinib plus Atezolizumab) phase 3 trial (Kelley et al., 2022), and KEYNOTE-524 phase 1b trial (Pembrolizumab plus Lenvatinib) (Finn et al., 2020b), and the LAUNCH phase 3 trial (Lenvatinib combined with transarterial chemoembolization) (Peng et al., 2022) have enhanced the overall survival and response rate. And the combination of immune checkpoint inhibitors and anti-angiogenic drug treatment has been authorized as a preferred first-line treatment for advanced HCC. However, only a small subset of HCC patients could benefit from this combination treatment.

With a further understanding, it has been realized that the tumor cells “do not act alone”, but interact directly or indirectly with stromal cells, immune cells, and non-cellular components (Fang and Declerck, 2013). Diverse environmental and genetic factors together regulated the HCC immune and angiogenesis microenvironment (Hou et al., 2020), which controls malignant progression and response to therapy. Up to now, the overall survival rate in the most effective IMbrave150 phase 3 trial was only 30%. And the phase III LEAP-002 study, immune checkpoint inhibitors combine with anti-angiogenic drug treatment (Lenvatinib plus pembrolizumab), as first-line therapy for advanced hepatocellular carcinoma had not achieved the expected results in advanced HCC (Finn1 et al., 2022). Therefore, a comprehensive analysis of the TIME components and anti-angiogenic subtypes pathways may predict and guide the combination treatment of immune and anti-angiogenic drugs, and find new therapeutic biomarkers (Binnewies et al., 2018).

m6A (N6-methyladenosine) is one of the most abundant RNA modifications in eukaryotic cells (He et al., 2019), which plays a considerable role in regulating the tumor immune microenvironment. As a dynamic reversible process, m6A modification is regulated through binding proteins, methyltransferases, and demethylases (Zaccara et al., 2019), also known as “readers”, “writers” and “erasers”. Furthermore, several studies have shown that the m6A-related signatures were associated with TIME in gastric cancer, colorectal cancer, low-grade glioma, as well as HCC (Zhang et al., 2020; Li et al., 2021a; Chong et al., 2021; Du et al., 2021). However, there were few studies focused on m6A-related signatures and the sensitivity of anti-angiogenic drugs.

In this study, we systematically analyzed 26 m6A regulators in HCC including the somatic mutation, copy number variation (CNV), and mRNA transcriptome. Besides, we identified three m6Aclusters with distinct immune and angiogenesis microenvironments and constructed a 5-gene prognosis signature (m6Asig-Score) based on m6A-related different genes (DEGs). We further found that two of five m6Asig-Score inner genes, B2M and SMOX, were associated with immune cell infiltration, immune response, and sensitivity to sorafenib. We validated these results in two independent immunotherapy cohorts and the Genomics of Drug Sensitivity in Cancer (GDSC) database. Understanding the interactions of m6A modification with immune and angiogenesis microenvironment was helpful to select the HCC patients who would benefit from immune and anti-angiogenic treatment.

## Data and methods

### HCC datasets collection and preprocessing

**FIGURE 1:**
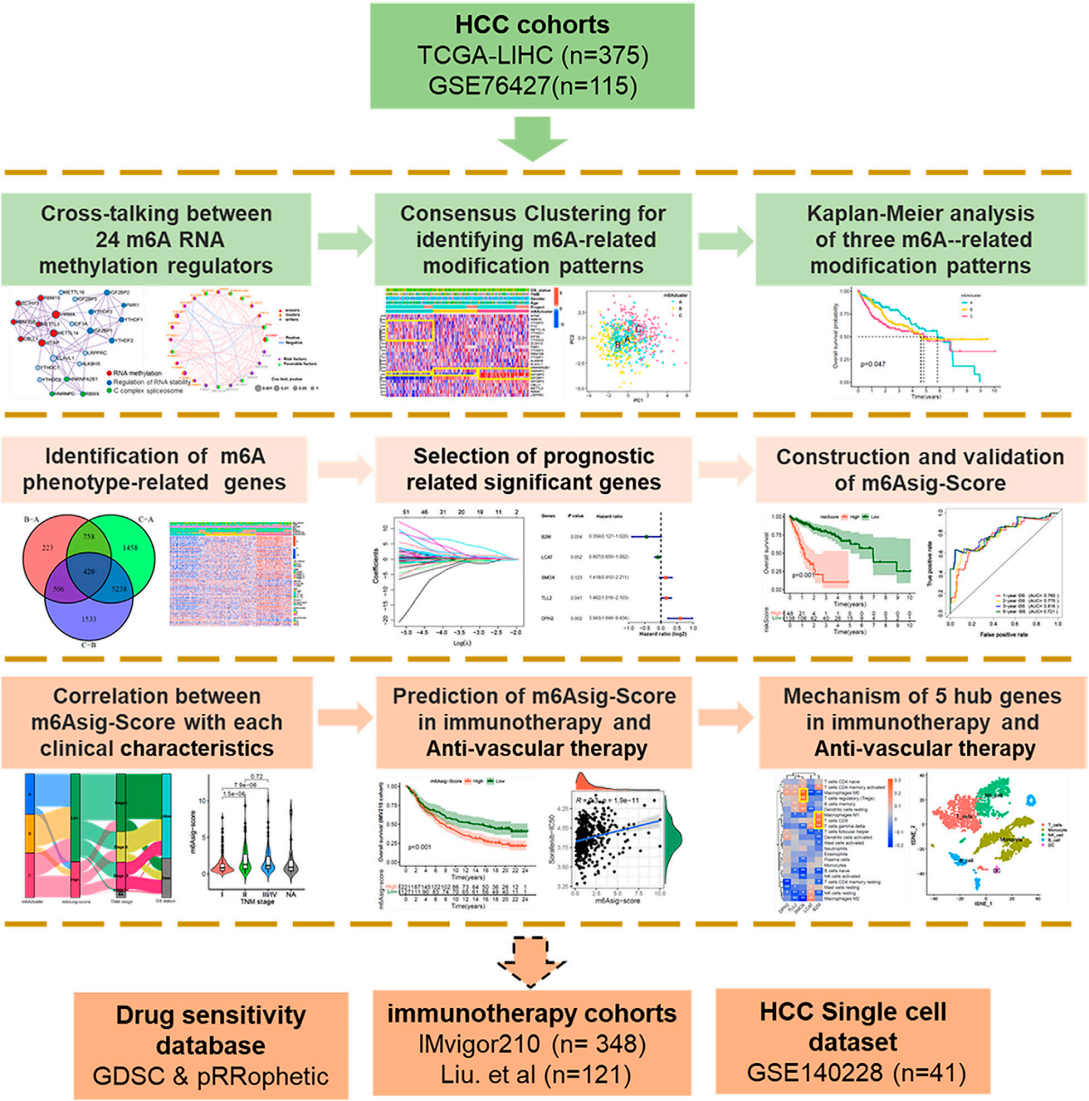
Overview of study design. A total of 490 HCC samples with completed transcriptome and survival information were concluded in this study. We first explored the multi-omics characteristic landscapes of 26 m6A regulators and identified three different m6Aclusters with distinct immune and angiogenesis phenotypes. Then, we further constructed a 5-genes prognosis signature termed as “m6Asig-Score” based on 420 m6A-related DEGs. The patients with high m6Asig-Score were associated with poor prognosis, advanced TNM stage and high TP53 mutation frequency. Finally, we explored the capability of m6Asig-Score and 5 hub genes in predicting immunotherapy and anti-angiogenic efficacy. Two independent immunotherapy cohorts (IMvigor210 and Liu. et al. cohorts) and the Genomics of Drug Sensitivity in Cancer (GDSC) database were used to validate the immunotherapy and anti-angiogenic efficacy respectively. And the HCC single immune cells sequencing datasets GSE140228 were performed to present the expression distribution of 5 hub genes.

The analysis workflow was shown in Figure 1. The copy number variation (CNV), somatic mutation, mRNA transcriptome, and clinical information of the TCGA-LIHC cohort were collected from Xena (https://xenabrowser.net). The mRNA expression and clinical information of the GSE76427 cohort were downloaded from the NCBI GEO database (https://www.ncbi.nlm.nih.gov/geo/). A total of 592 HCC samples were included in this study, including those from TCGA-LIHC cohort (non-tumor = 50, tumor = 375) and GSE76427 dataset (non-tumor = 52, tumor = 115). The RNA Transcriptome data (FPKM format) were converted to the transcript format of millions per kilobase (TPM). The operational functions in the “SVA” package were employed to eliminate the batch effect between the GSE76427 queue and the TCGA-LIHC dataset (Yang et al., 2020). The “Rcircos” package was suitable for drawing the CNV diagram of 26 m6A regulators in HCC.

### Consensus molecular clustering of 24 m6A regulators by PAM

Based on published literature, we curated 26 acknowledged m6A regulators and analyzed them to identify divergent m6A-related patterns. The 26 m6A regulators included 15 readers (EIF3A, ELAVL1, HNRNPA2B1, HNRNPC, FMR1, IGF2BP1-3, YTHDC1/2, YTHDF1-3, LRPPRC, RBMX), 9 writers (CBLL1, METTL3/14/16, RBM15/15B, VIRMA, WTAP, and ZC3H13) and 2 erasers (ALKBH5 and FTO) (Meyer et al., 2015; Chen et al., 2020; Shulman and Stern-Ginossar, 2020; Li et al., 2021b). Among these 26 m6A regulators, METTL16 and VIRMA were not annotated in the GSE76427 cohort. Based on the expression of 24 overlapping regulators in the combined HCC cohort (GSE76427 and TCGA-LIHC), we performed a consistent cluster analysis of medoid (PAM) to determine different m6A-related modification patterns. The number and stability of clusters were determined by the R package “ConsensusClusterPlus”, and conducted for 1,000 times repetitions (Seiler et al., 2010; Wilkerson and Hayes, 2010).

### Gene set variation analysis of KEGG pathways and calculation of VEGFR score

Gene set variation analysis (GSVA) algorithm was applied for investigating pathway variations among different m6A modification patterns *via* the “GSVA” package (Hanzelmann et al., 2013). The KEGG pathways in the MSigDB database (http://www.gsea-msigdb.org) were downloaded for GSVA analysis. The genes VEGR1-3 are the targets of anti-angiogenic drugs Regorafenib, Lenvatinib, Sorafenib, and Donafenib, which may indicate the therapeutic effect. And the VEGFR score was estimated by Gene set variation analysis (GSVA) based on VEGR1-3 gene expression.

### Estimation of immune cell infiltration by ssGSEA, MCPCOUNT, and TIMER 2.0 algorithm

The fraction of infiltration immune cells was evaluated by the single-sample GSEA (ssGSEA) algorithm *via* the “GSVA” package, in which 28 immune cell types were identified by specific feature gene markers curated from previous studies (Charoentong et al., 2017; Jia et al., 2018). The ssGSEA enrichment score represents the relative abundance of each type of immune cell, and the unit distribution is normalized from 0 to 1. MCPCOUNT was employed to estimate the fraction of endothelial cells, fibroblasts, and 8 immune cell subsets with the mRNA transcriptome profiles (Becht et al., 2016). Tumor Immune Estimation Resource (TIMER 2.0; cistrome.shinyapps.io/timer) was used to estimate the molecular features of 6 immune cell subsets (Li et al., 2017).

### Identification of m6A-related different expression genes

The previous algorithm “PAM” was employed to classify HCC patients into three different m6A modification clusters. The “limma” package was performed to evaluate DEGs in HCC samples, and the “heatmap” package was used for describing the expression landscape of DEGs among three different m6A clusters. Adjusted *p* value < 0.001 was considered the significant criterion for identifying DEGs.

### Construction of a prognosis signature based on m6A-related DEGs

A total of 370 TCGA-LIHC samples with completed survival information were randomly divided into the TCGA training and TCGA testing cohorts *via* the “caret” package. The mRNA expression of 420 m6A-related DEGs was extracted from the TCGA training, TCGA testing, and GSE76427 cohorts. The process and outcome of the univariate-Cox, lasso-Cox, and multivariate-Cox regression analysis were performed *via* “survival” and “glmnet” packages. The m6A-related DEGs with a remarkable prognostic value (*p* < 0.05) were filtrated. In the multivariate Cox regression analysis, the m6A-related prognosis signature (termed as m6Asig-Score) was expressed as follows: m6Asig-Score = $\overset{n}{\underset{i}{\sum }}coef_{i}*mRNA_{i}$ . The optimal cutoff value of m6Asig-Score was selected by the “surv_cutpoint” function of “survival” package, and the patients in the TCGA training cohort were classified into the high- and low-m6Asig-Score groups. The TCGA testing and GSE76427 cohorts were also assigned into high- and low-m6Asig-Score groups by the same cutoff value. The Kaplan–Meier survival curves and time-ROC curves were performed *via* “survminer” and “survivalROC” packages.

### Genomic and clinical data of the immunotherapeutic cohorts

We systematically explored the publicly obtained immunotherapeutic cohort, which contained gene expression profiles and integral clinical information. Two immunotherapeutic cohorts (IMvigor210 cohort and Liu et al. cohort) were finally enrolled in this study. The IMvigor210 cohort (Mariathasan et al., 2018) contained 348 metastatic urothelial carcinoma patients with Atezolizumab (anti-PD-L1 mAb) therapy and Liu et al. cohort (Liu et al., 2019) incorporated 121 melanoma patients with anti-PD-1/PD-L1 therapy. The transcriptome profiles of two immunotherapeutic cohorts were transformed into the TPM before further analysis.

### Distribution of 5 hub genes in HCC single immune cells sequencing dataset

The GSE140228 cohort (Zhang et al., 2019)contained single-cell RNA sequencing of CD45^+^ immune cells from tumor tissue, peritumoral normal liver tissue, blood, and ascites in HCC patients. The corresponding sequencing data and the sample annotation information were downloaded in “https://www.ncbi.nlm.nih.gov/geo/”. The packages “Celldex” and “SingleR” (Aran et al., 2019) were used to annotate cell types based on reference cell markers, and to present the expression landscapes of 5 hub genes in different sites and immune cell subpopulations of HCC patients.

### Estimation of anti-angiogenic drugs IC_50_ and the immune response biomarkers: IPS, TIS and TIDE

To explore the prediction of m6Asig-Score in anti-angiogenic drugs sensitivity. We performed Spearman correlation analysis between m6Asig-Score and the IC_50_ of Sorafenib, Erlotinib, Lapatinib, Dasatinib, and Pazopanib. The IC_50_ of anti-angiogenic drugs in GDSC (Genomics of Drug Sensitivity in Cancer) database, which contained a large panel of cancer cell lines, was calculated by the “pRRophetic” package (Geeleher et al., 2014). Immunophenoscore (IPS) is a powerful biomarker for predicting immunotherapy response, which was used to estimate the determinants of tumor immunogenicity based on four panels of immune-related molecules: MHC, immune checkpoints, effector cells and suppressor cells (Charoentong et al., 2017). The IPS data of TCGA-LIHC cohort were downloaded from the Cancer Immunome Atlas (https://tcia.at/). Tumor Inflammation Signature (TIS), an 18-gene signature, which symbolizes the presence of a suppressed adaptive immune response, was evaluated to predict anti-PD-1(pembrolizumab) therapy benefit, (antigen presentation, and IFN gamma and cytotoxic cells) in the TME (Danaher et al., 2018). In addition, Tumor Immune Dysfunction and Exclusion (TIDE) was applied to survey two different tumor immune escape mechanisms, cytotoxic T lymphocytes (CTLs) dysfunction and immunosuppressive factor rejection of CTL (Jiang et al., 2018). Patients with higher TIDE scores were more likely to escape anti-tumor immunity, thereby achieving lower effectiveness of Immunotherapy.

### Statistical analyses

This study performed R-4.0.2 for statistical analyses. Student’s t-test or Wilcoxon rank-sum test were used to estimating the statistical significance of two groups’ comparisons (normally or non-normally distributed variables). For comparisons among three groups, the one-way ANOVA analysis and Kruskal–Wallis tests were applied as nonparametric and parametric methods (Hazra and Gogtay, 2016). Kaplan-Meier survival analysis was utilized to explain the prognosis association of distinct m6A modification patterns, m6Asig-Score, and the 5 hub genes expression. The mutation landscape of two m6Asig-Score subgroups was presented through the R package “maftools” (Mayakonda et al., 2018). The CNV landscape in human chromosomes of 26 m6A regulators was adopted by the “RCircos” package (Zhang et al., 2013). The test was bilateral, and *p* < 0.05 was considered significant, and the adjusted *p*-value (FDR, false discovery rate) was used for multi hypothesis test (Ferreira, 2007).

## Results

### Multi-omics characteristic landscapes of 26 m6A regulators in HCC

**FIGURE 2:**
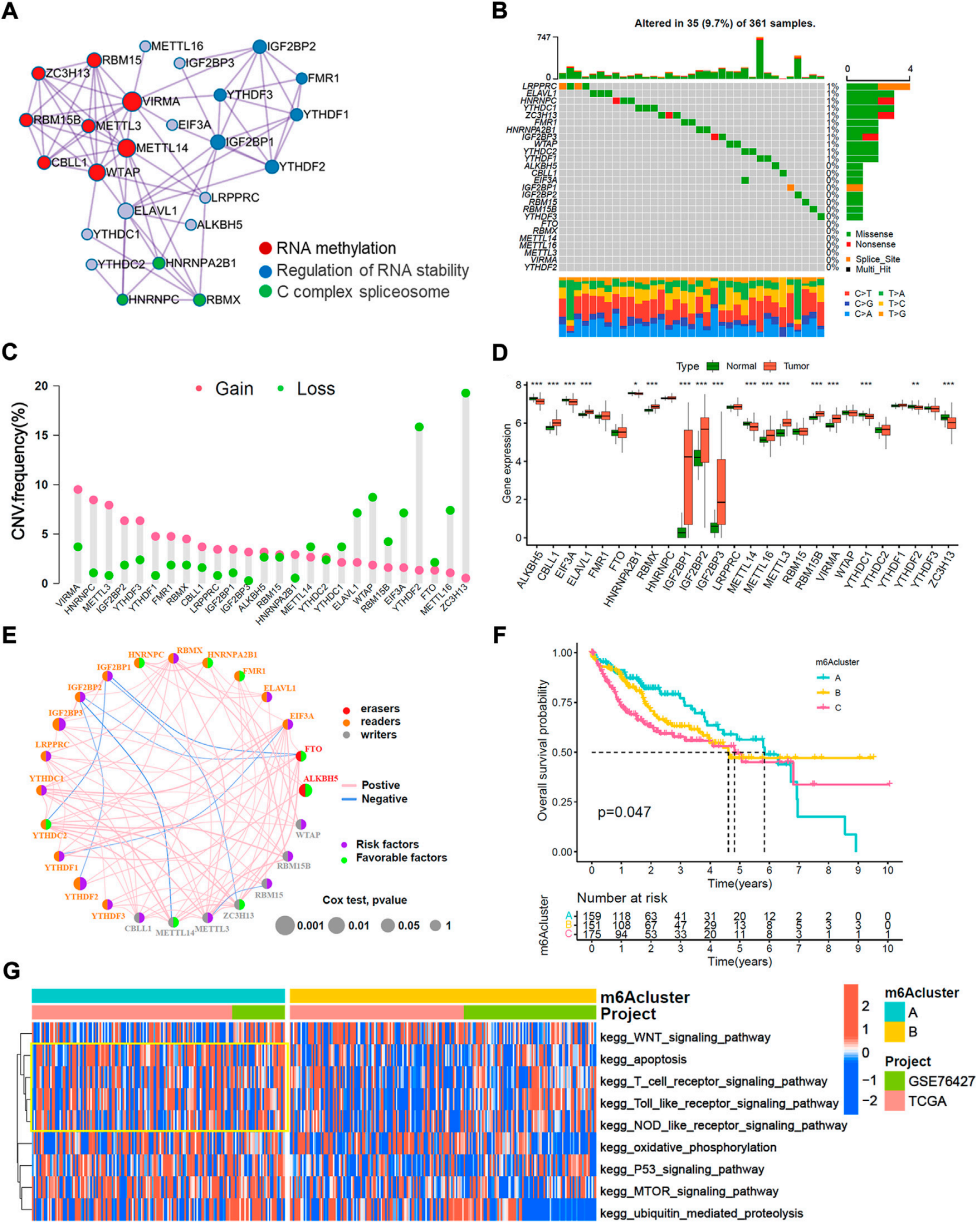
Multi-omics characteristic landscapes of 26 m6A regulators and relevant biological pathway of m6A modification pattern in HCC. **(A)** The intra-clusters similarities of 26 m6A regulators were visualized by the metascape enrichment network, and the cluster annotations were presented with color code, including methylation, stability, and C complex spliceosome. **(B)** Genetic mutations landscape of 26 m6A regulators was found in 35of the 361 HCC patients, with a frequency of 9.7%, and the mutation frequency of each regulator were shown on the right. **(C)** The bar plot indicated the CNV alterations frequency of regulators in the TCGA-LIHC cohort. The amplification frequency was presented in pink dot, and the deletion frequency was in green dot. **(D)** The transcriptome landscape of regulators between normal and HCC samples in TCGA-LIHC cohort. The statistical *p*-value was showed by the asterisks above (ns, *p* > 0.05; *p < 0.05; **p < 0.01; ***p < 0.001). **(E)** The interaction of expression on 24 m6A regulators were sequenced in both TCGA and GSE76427 HCC cohorts. The 24 m6A regulators were depicted into three RNA modification types by the left half of circle in different colors. Erasers, red, Readers, yellow; Writers, blue; and the lines connecting each m6A regulator represented their interaction with each other. The size of each circle was referring to the overall survival (OS) *p*–value. The green dot on the right half of circle indicated favorable factors for HCC prognosis, while the purple dot represented risk factors. **(F)** Kaplan-Meier curves of overall survival (OS) for merged HCC cohorts (TCGA-LIHC and GSE76427 cohorts) among three distinct m6A clusters. The numbers of patients in m6Aclusters A, B and C phenotypes are 159, 151, and 175, respectively (Log-rank test, *p* < 0.05). **(G)** Heatmap indicated the significant KEGG pathways curated from MSigDB in m6Acluster A vs. m6Acluster B.

The overview of this work was shown in Figure 1. In this study, we determined 26 m6A regulators, including 15 readers (EIF3A, ELAVL1, FMR1, HNRNPA2B1, HNRNPC, IGF2BP1-3, LRPPRC, RBMX, YTHDC1/2, YTHDF1-3), 9 writers (CBLL1, METTL3/14/16, RBM15/15B, VIRMA, WTAP and ZC3H13) and 2 erasers (ALKBH5 and FTO). Metascape analyses showed that 26 m6A regulators were markedly enriched in RNA methylation, RNA stability, and C-complex spliceosome pathways (Figure 2A). In the TCGA-LIHC cohort, we explored the somatic mutation landscape of 26 m6A regulators. The results showed that 35 of 361 (9.7%) HCC samples experienced somatic mutations, primarily including missense mutations, nonsense mutations, and splice site mutations (Figure 2B). The CNV landscape of 26 m6A regulators revealed that VIRMA, HNRNPC, METTL3, IGF2BP2, and YTHDF3 showed widespread CNV amplification, while ZCH13, YTHDF2, WTAP, ELAVL1, METTL16, and EIF3A presented a tendency to CNV deletions (Figure 2C). Further analysis demonstrated that IGF2BP1-3, METTL16, METTL3, RBMX, RBM15B, and VIRMA were significantly upregulated, whereas ALKBH5, CBLL1, EIF3A, METTL14, YTHDC1, and ZC3H13 were significantly downregulated (Figure 2D). We further explored the association between 26 m6A regulators and the prognosis of HCC patients through univariate Cox analysis. The forest-plot of disease-free survival (DFS) and overall survival (OS) showed that the ALKBH5 was significantly associated with better DFS and OS and could be recognized as a protective factor, while the IGF2BP2 was a risk factor and related to worse DFS and OS (Supplementary Figures S1A,B). The above analyses demonstrated a high heterogeneity of the multi-omics alteration landscape for 26 m6A regulators, which presented the crucial role of m6A regulators in HCC relapse and progression.

### Identification of m6A-related modification patterns and relevant biological pathway

TCGA-LIHC cohort and GSE76427 cohort with available survival data were enrolled in this study. Among 26 m6A regulators, METTL16 and VIRMA were not annotated in the GSE76427 cohort. The comprehensive interaction network of 24 m6A regulators illustrated the prognostic significance and their cross-talks in HCC patients (Figure 2E). Furthermore, we performed a consistent cluster analysis of medoid (PAM) and determined three disparate m6A-related modification patterns based on the expression of 24 m6A regulators (Supplementary Figures S2A–E). Three m6A-related modification patterns termed as the m6Acluster-A (n = 159), m6Acluster-B (n = 151) and m6Acluster-C (n = 175). The patients in m6Acluster-A dominated better overall survival, while the m6Acluster-C had the worst prognosis (*p* = 0.047, log-rank test). Moreover, the principal component analysis (PCA) demonstrated that m6A-related patterns were completely distinguished among three distinct m6Aclusters (Supplementary Figure S2F). We also noticed that YTHDC2, and ZC3H13 were significantly increased in the m6Acluster-A subtype; IGF2BP1, IGF2BP2, IGF2BP3, CBLL1, RBMX, METTL3 and RBM15B were significantly elevated in the m6Acluster-C subtype; and the m6Acluster-B subtype shared intermediate expression in most 26 m6A regulators (Supplementary Figure S3A). We further explored the biological behaviors of three distinct m6A-related patterns *via* GSVA analysis. The heatmap showed that m6Acluster-A enriched with immune activation including T cell receptor, NOD-like receptor, and Toll-like receptor signaling pathways (Figure 2G). The m6Acluster-B associated metabolic reprogramming pathways such as the PPAR signaling pathway and glycerolipid metabolism pathway (Supplementary Figure S3B). Whereas the m6Acluster-C was prominently related to the cell cycle pathway and base excision pathway (Supplementary Figure S3C).

### Immune and angiogenesis microenvironment characteristics in three m6A-related modification patterns

**FIGURE 3:**
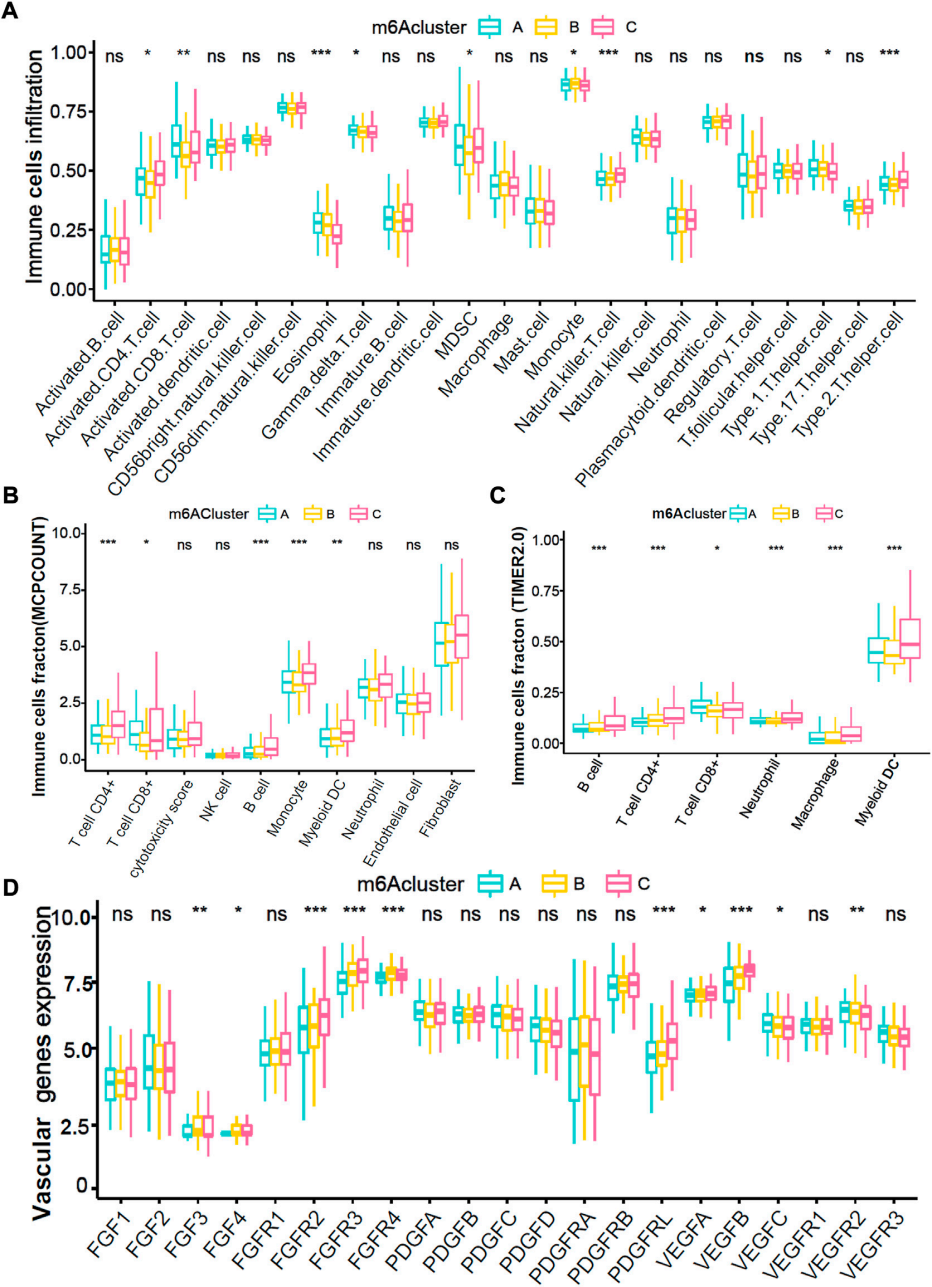
Immune and angiogenesis microenvironment characteristics in distinct m6A modification patterns. **(A)** The fraction of TME cells in three distinct m6A-related patterns. **(B**,**C)** The abundance of tumor-infiltrating immune cells in three m6A-related patterns were calculated *via* MCPCOUNT and TIMER2.0 algorithm. B, CIBERSORT; C, TIMER2.0. **(D)** The abundance of each 21 vascular-related gene expression in three m6A-related patterns. The top and bottom of the boxes manifested the value of the interquartile range, and the lines in the boxes represented median value. The fraction of TME infiltrating cells among three m6Acluster were compared *via* the Kruskal–Wallis H test, and gene expression difference was compared *via* the one-way ANOVA test.

Immune cell infiltration analyses demonstrated that the m6Acluster-A enriched active immune cells, such as CD8^+^ T cells and eosinophils. The m6Acluster-C was enriched in innate immune cells, such as CD4^+^ T cell, Type 2 T helper cell, and myeloid dendritic cell, but lower in eosinophil (Figure 3A). We further confirmed the immune cell infiltration characteristics with MCPCOUNT and TIMER2.0. The CD4^+^ T cell, monocyte/macrophage, and myeloid dendritic cell were remarkably enriched in m6Acluster-C (Figures 3B,C). Angiogenesis microenvironment characteristics analyses showed that FGFR-related and VEGF-related genes expression were significantly enhanced in m6Acluster-C (Figure 3D). However, the patients in m6Acluster-C did not exhibit a matching prognostic benefit in Figure 2F. The previous study showed that tumors with immune exclusion phenotypes also showed abundant immune cells distributed in the stroma circumambient tumor cell nests (Chen and Mellman, 2017). Therefore, we assumed that stromal and angiogenesis activation in m6Acluster-C may inhibit the anti-tumor effect of immune cells.

### Construction of a prognosis signature base on m6A-related DEGs

**FIGURE 4:**
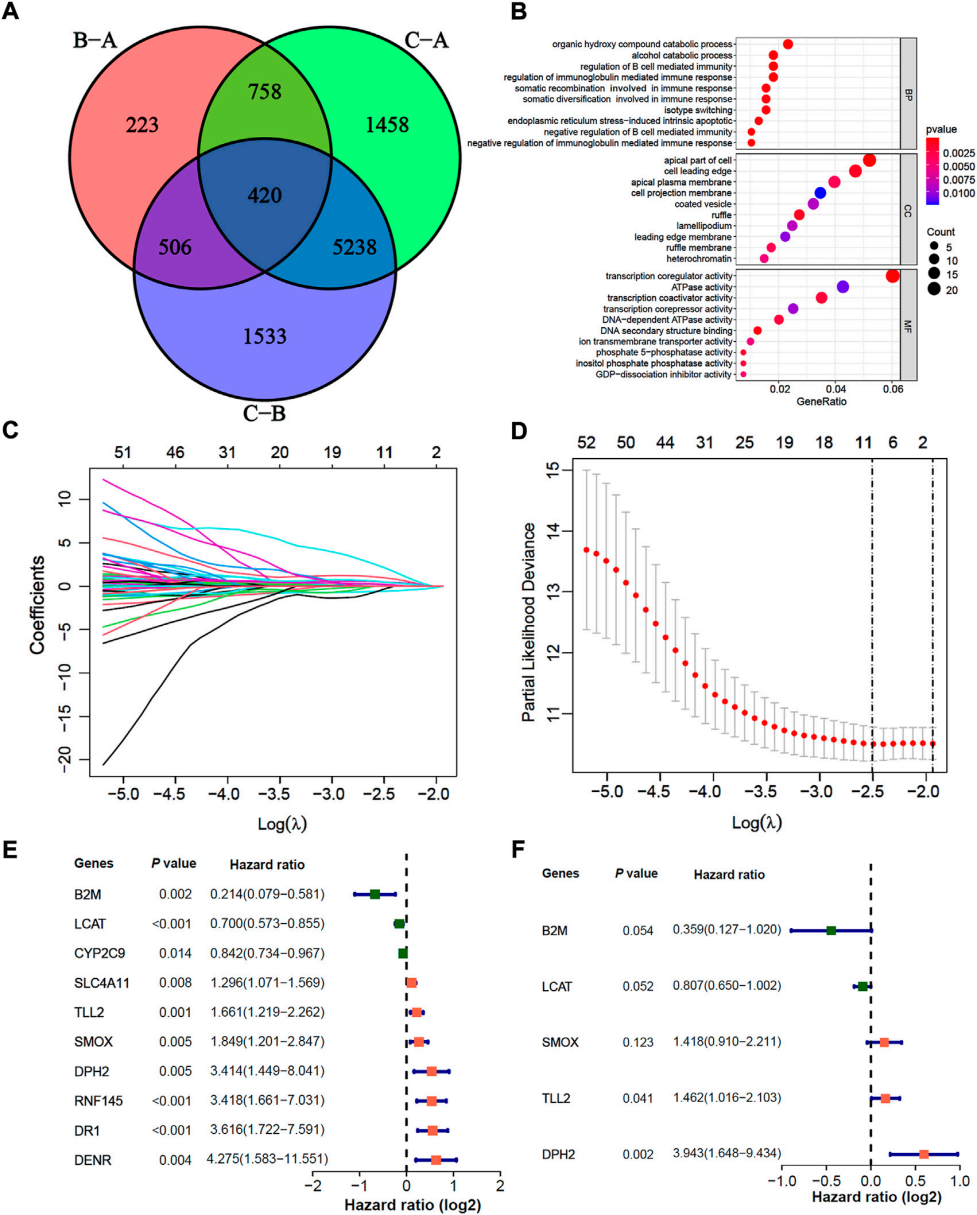
Construction of a prognosis signature based on m6A-related differential expression genes. **(A)** The Venn diagram indicated 420 m6A-related DEGs among three m6A-related clusters. **(B)** GO enrichment analysis for 137 m6A-related and prognosis significant DEGs. The dot size indicated the counts of genes enriched, and the dot color manifested the *p* value. **(C**,**D)** Lasso coefficient profiles and optimal parameter (lambda) selection used 5-fold cross-validation. A coefficient profile plot was produced against the log(lambda) sequence. The partial likelihood deviance (binomial deviance) curve was plotted versus log(lambda). Dotted vertical lines were drawn at the optimal values by using the minimum criteria and the 1-SE of the minimum criteria. Abbreviations: SE, standard error. **(E**,**F)** The Cox forest-plots of 10 Lasso genes and 5 multivariate Cox genes in the TCGA-LIHC training cohort.

In total, we identified 420 significant m6A-related DEGs (different expression genes) and as shown in the Venn diagram (Figure 4A, adjusted *p* < 0.05). We further explored the GO enrichment analysis, and the result showed that 420 DEGs were mostly enriched in immune response pathways (Figure 4B). In the TCGA-LIHC training cohort, 61 of 420 DEGs were selected through univariate Cox, and 10 of 61 DEGs were further screened by the lasso-Cox regression algorithm (Figures 4C,D). Finally, 5 prognostic-related hub DEGs were identified by multivariate Cox regression analysis, The forest-plot showed the hazard ratio of 10 lasso genes and 5 hub genes (Figures 4E,F). Kaplan-Meier analysis indicated that the patients with high expression of B2M and LCAT were significantly related to poor prognosis (Supplementary Figures S4A,D; both *p* < 0.05, Log-rank test), while high expression of DPH2, SMOX and TLL2 indicated better prognosis (Supplementary Figures S4G,J,M; all *p* < 0.05, Log-rank test). And the prognosis value of B2M, DPH2 and TLL2 were confirmed in the TCGA-LIHC testing cohort and GSE76427 cohort (Supplementary Figures S4B,C,H,I,K,L; all *p* < 0.05, Log-rank test). While the prognosis of LCAT in GSE76427 cohort (Supplementary Figure S4F; *p* = 0.462, Log-rank test) and TLL2 in TCGA-LIHC testing (Supplementary Figure S4N; *p* = 0.680, Log-rank test) and GSE76427 cohorts (Supplementary Figure S4O; *p* = 0.158, Log-rank test) did not have a significant difference, which may due to tumor heterogeneity and the samples selection differences.

**FIGURE 5:**
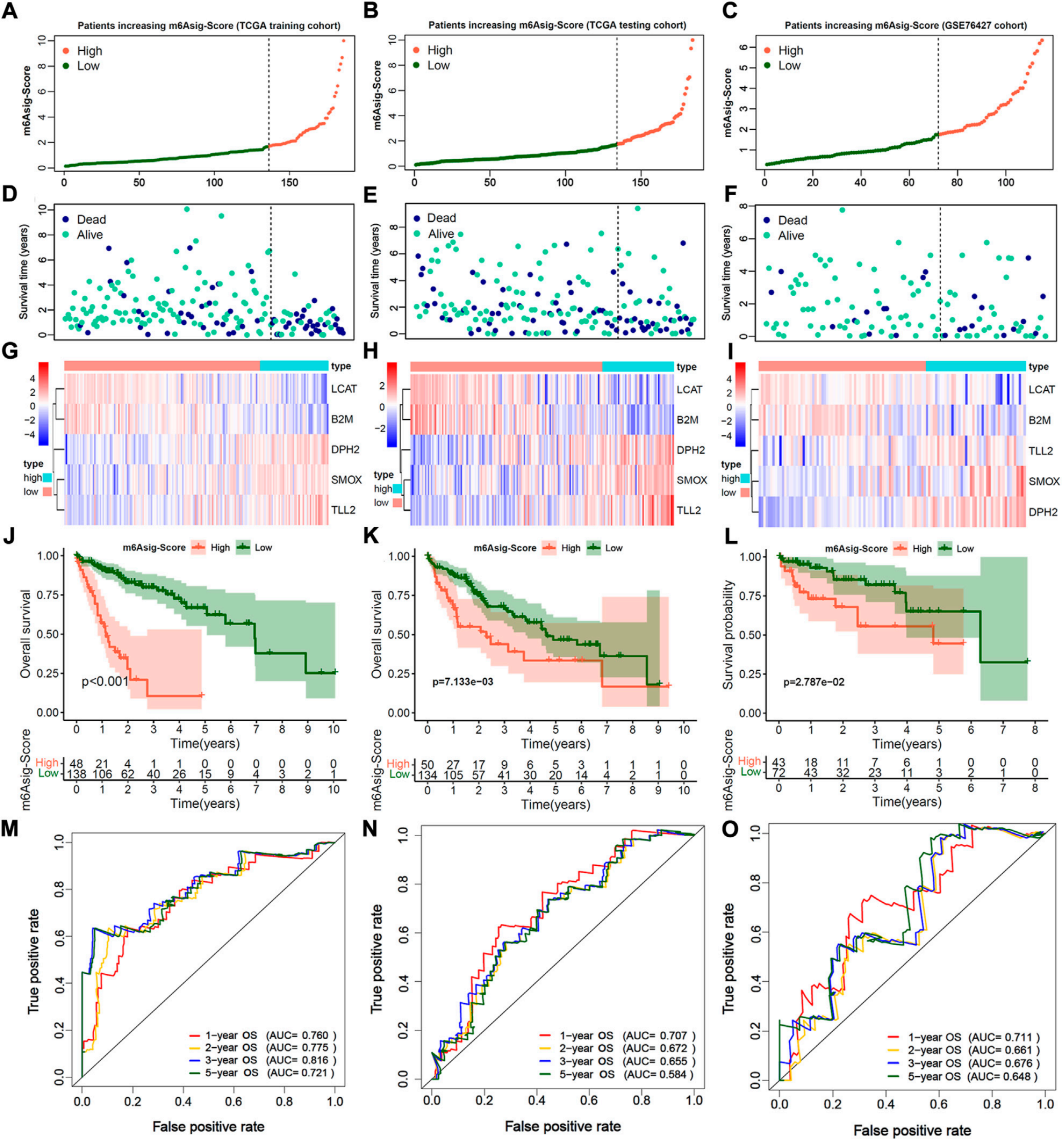
Estimation and validation of m6A significant score (m6Asig-Score). **(A**–**C)** The assessment of risk for m6Asig-Score in three distinct HCC cohorts, different colors of curves represented risk score. High risk was presented in tomato, and low risk was in green. **(D**–**F)** The plots of different colors and locations represented OS status and survival time in three HCC distinct cohorts. Blue, dead; green, alive. **(G**–**I)** The heatmap indicates 5 hub genes expression between high and low m6Asig-Score in three distinct HCC cohorts. **(J**–**L)** Kaplan-Meier analysis of overall survival (OS) for high and low m6Asig-Score groups in three distinct HCC cohorts. **(M**–**O)** Time-ROC curves of 1-, 2-,3-, and 5-year OS in three distinct HCC cohorts.

We further constructed a prognosis signature termed as m6Asig-Score to predict the prognostic risk of HCC (details in Methods). The m6Asig-Score, survival time, alive status and the heatmap of 5 hub genes expression of HCC were exhibited in Figures 5A–I. The patients with increasing m6Asig-Score had a high fraction of death status, and high expression of DPH2, SMOX, TLL2, AND low expression of LCAT and B2M. The optimal cutoff value of m6Asig-Score was selected by the “surv_cutpoint” function of “survival” package, and the patients in the TCGA-LIHC training, TCGA-LIHC testing, and GSE76427 cohorts were classified into high m6Asig-Score and low m6Asig-Score groups. In the TCGA-LIHC training cohort, the Kaplan-Meier survival analysis indicated patients with low m6Asig-Score were probably led to a better prognosis than patients with low m6Asig-Score (Figure 5J, *p* < 0.001, Log-rank test), and the result was confirmed in TCGA-LIHC testing and GSE76427 cohorts (Figures 5K,L, both *p* < 0.001, log-rank test). In the TCGA-LIHC training cohort, the ROC curve analysis exhibited that the AUC values of 1-, 2-,3-, and 5-year survival was 0.760, 0.775, 0.816, and 0.721, respectively (Figure 5M). And similar results were found in TCGA-LIHC testing and GSE76427 validation cohorts (Figures 5N,O).

### Relevance exploration of m6Asig-Score with clinical features and tumor somatic mutation

**FIGURE 6:**
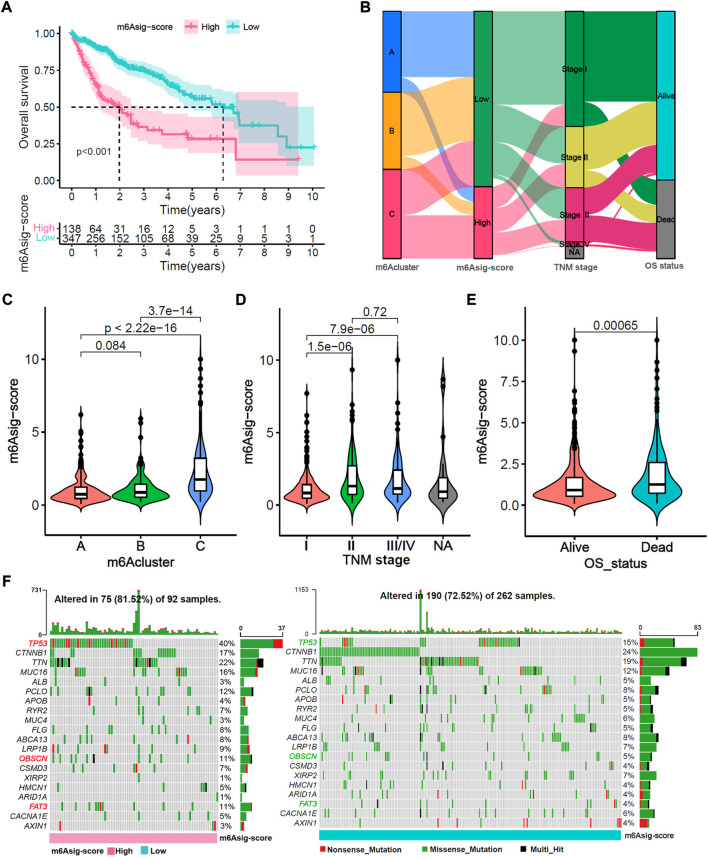
Relevance exploration of m6Asig-Score with clinical features and tumor somatic mutation. **(A)** Overall survival Kaplan-Meier analysis for high and low m6Asig-Score groups in merged HCC cohort (*p* < 0.001, Log-rank test). **(B)** The Alluvial diagram explained the analysis workflow of m6Acluster, m6Asig-Score, TNM stages and survival status. **(C**–**E)** The m6Asig-Score was compared among distinct m6Acluster, TNM stage and OS status. **(F)** The tumor somatic mutation waterfall between high m6Asig-Score (left) and low m6Asig-Score (right) subgroup in the TCGA-LIHC cohort. The upper number of the bar plot indicated tumor mutation burden (TMB), and the right number showed mutation frequency.

A total of 485 HCC patients were classified into the high m6Asig-Score and low m6Asig-Score groups based on the optimal cutoff value of m6Asig-Score (high group includes 138 patients and low group includes 347 patients). The patients with low m6Asig-Score led a better prognosis than patients with high m6Asig-Score in the total cohort as well as in different age (60 and above) and TNM stage (I/II and III/IV) subgroups (Figure 6A, Supplementary Figures S5A–D; all *p* < 0.001, Log-rank test). The alluvial diagram explained the analysis workflow of m6Aclusters, m6Asig-Score, TNM stage, and survival status (Figure 6B). And the consequences showed that m6Acluster-C exhibited a higher m6Asig-Score, advanced TNM stage and higher dead risk. Nevertheless, m6ACluster-A was associated with lower m6Asig-Score, early TNM stage and alive status. The m6Acluster-C exhibited the m6Asig-Score (Figure 6C, *p* < 0.001, Wilcoxon rank-sum test). Besides, the m6Asig-Score of patients in TNM stage II and III/IV was significantly higher than patients in TNM stage I (Figure 6D, *p* < 0.001, Wilcoxon test), and the m6Asig-Score of patients in dead status was also significantly higher than patients in alive status (Figure 6E, *p* < 0.001, Wilcoxon rank-sum test). The somatic mutation landscapes illustrated that the somatic mutation rates of TP53 (40% vs. 15%), OBSCN (11% vs. 5%) and FAT3(11% vs. 4%) were significantly higher in the high m6Asig-Score subgroup than in low m6Asig-Score subgroup (Figure 6F, *p* < 0.05, Fisher’s exact test). These data indicated the potentially complex interaction between the prognosis signature m6Asig-Score and HCC somatic mutations.

We further evaluated the TIME cell infiltration between two m6Asig-Score subgroups. The low m6Asig-Score subgroup was remarkably enriched in adaptive immune cells, such as activated CD8^+^ T cell, activated B cell, eosinophil, natural killer cell and Th 1 cell. While the high m6Asig-Score group was enriched in innate immune cells, such as CD4^+^ T cells, immature dendritic cells and Th 2 cells (Supplementary Figure S6A). Furthermore, we compared the RNA expression levels of 21 vascular-related genes between two m6Asig-Score subgroups. In the low m6Asig-Score subgroup, the VEGFR1-3 were significantly highly expressed, while the FGFR 1, FGFR 3, PDGFRL, VEGFA, and VEGFB were significantly low expressed (Supplementary Figure S6B). The results were confirmed in the correlation between m6Asig-Score and known TME signatures and the expression of vascular-related genes (Supplementary Figures S6C,D).

### The potential of m6Asig-Score in predicting immunotherapeutic and anti-angiogenic therapy response

**FIGURE 7:**
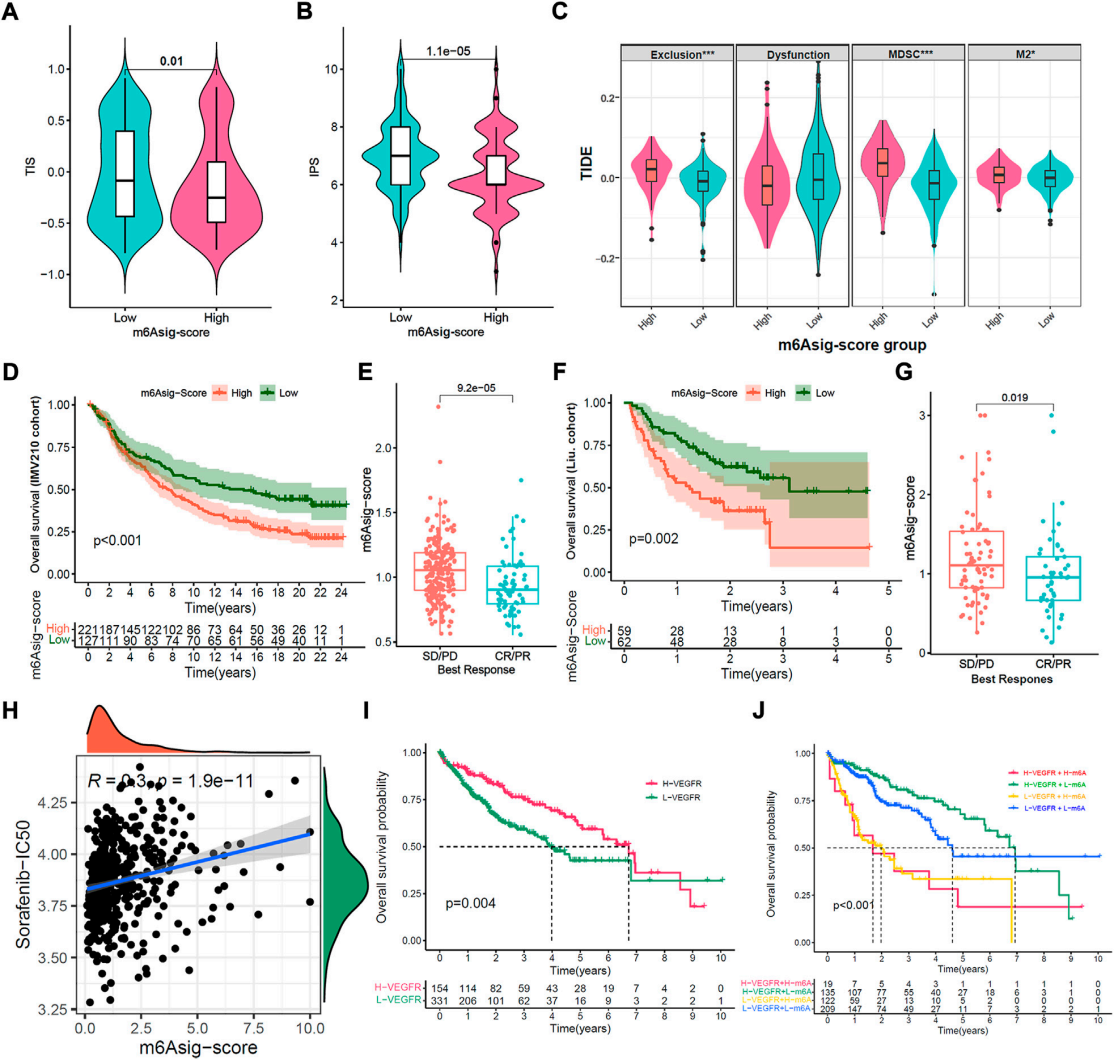
Prediction of m6Asig-Score in immunotherapy and anti-angiogenic therapy. **(A**–**C)** The relative distribution comparison of TIS **(A)**, IPS **(B)** and TIDE **(C)** between high m6Asig-Score and low m6Asig-Score groups. **(D)** Kaplan-Meier analysis between high m6Asig-Score and low m6Asig-Score groups in IMvigor210 cohort (*p* < 0.001, Log-rank test). **(E)** The comparison of m6Asig-Score between SD/PD and CR/PR two groups in IMvigor210 cohort. **(F)** Kaplan-Meier analysis between high m6Asig-Score and low m6Asig-Score groups in Liu. cohort (*p* = 0.002, Log-rank test) **(G)** The comparison of m6Asig-Score between SD/PD and CR/PR two groups in Liu. cohort. **(H)** Spearman correlations analysis between sorafenib-IC_50_ and m6Asig-Score. **(I)** Overall survival Kaplan-Meier curves for high VEGFR-score and low VEGFR-score groups in the merged HCC cohort (*p* = 0.003, Log-rank test). **(J)** Kaplan-Meier survival analyses for patients stratified by m6Asig-Score and VEGFR-score. H, high; L, Low; Ve., VEGFR; m6A., m6Asig-Score (*p* < 0.0001, Log-rank test).

ICIs treatments represented by PD-1/PD-L1 inhibitors have made a great break in cancer treatment. As well as PD-L1, TMB, and MSI (Chen et al., 2019a; Chen et al., 2019b), the IPS, TIS, and TIDE were recently identified and widely applied to predict the immune response (Charoentong et al., 2017; Danaher et al., 2018; Jiang et al., 2018). Likewise, our study indicated that the TIS and IPS were markedly elevated in the low m6Asig-Score subgroup, and TIDE (Exclusion, MDSC, and M2) was prominently decreased in the low m6Asig-Score subgroup (Figures 7A–C, all *p* < 0.001). Furthermore, we investigated the capability of m6Asig-Score in predicting patients’ response to immunotherapeutic in two independent cohorts (IMvigor210 cohort and Liu et al. cohort). The results showed that patients in the IMvigor210 cohort with low m6Asig-Score presented significant prolong overall survival probability (Figure 7D, *p* < 0.001, Log-rank test), and the m6Asig-Score was significantly higher in CR/PR (complete response or partial response) patients than that in SD/PD (stable disease or progressive disease) patients (Figure 7E, *p* < 0.001, Wilcoxon test). And similar results were also found in Liu et al. cohort (Figure 7F, *p* < 0.001, log-rank test; Figure 7G, *p* = 0.019, Wilcoxon test).

To further explore the sensitivity prediction of m6Asig-Score in anti-angiogenic drugs. We performed Spearman correlation analysis between m6Asig-Score and the IC_50_ of Sorafenib and Pazopanib. The outcome indicated that m6Asig-Score was significantly positively correlated with the IC_50_ of Sorafenib and Pazopanib (Figure 7H and Supplementary Figure S7A, both *p* < 0.05, Spearman correlation test). This finding indicated that patients with low m6Asig-Score, were more likely to benefit from anti-angiogenic therapy.

We further explored the correlation between 5 hub genes and Sorafenib targeted genes. The heatmap showed that B2M significantly correlated with VEGFR1/2, while SMOX negatively correlated with VEGFR1-3 (Supplementary Figure S7B, *p* < 0.01, Spearman correlation test). The VEGR1-3 are the targets of anti-angiogenic drugs such as Sorafenib, Regorafenib and Lenvatinib, which may indicate the therapeutic effect. We further explored the correlation between vascular-related gene signatures and the IC_50_ of anti-angiogenic drugs. Our results revealed that m6Asig-Score significantly negatively correlated with the angiogenesis and VEGFR signatures, and positively correlated with the FGFR signature (Supplementary Figure S8A). And the VEGR and FGF signatures were significantly negatively correlated with the IC_50_ of Sorafenib (Supplementary Figures S8B,C; all *p* < 0.05, Spearman correlation test). The PDGFR and VEGF signatures significantly positively correlated with the IC_50_ of Sorafenib (Supplementary Figures S8D,E; all *p* < 0.05, Spearman correlation test). The FGFR, PDGF and angiogenesis signatures have no significant correlation (Supplementary Figures S8F–H; all *p* < 0.05, Spearman correlation test). These findings indicated that the VEGFR signature can predict the efficacy of the anti-angiogenic drug Sorafenib therapeutic. As expected, we found that the patients with high VEGFR signature presented a prolonged survival time (Figure 7I, *p* = 0.004, Log-rank test), and the patients with both high VEGFR signature and low m6Asig-Score exhibited a prominent survival benefit (Figure 7J, *p* < 0.001, Log-rank test). To sum up, our findings convincingly demonstrated that the m6Asig-Score was a powerful biomarker for predicting the immunotherapeutic and anti-angiogenic responses to HCC.

### The potential mechanism of 5 hub genes in immunotherapeutic and anti-angiogenic therapy.

**FIGURE 8:**
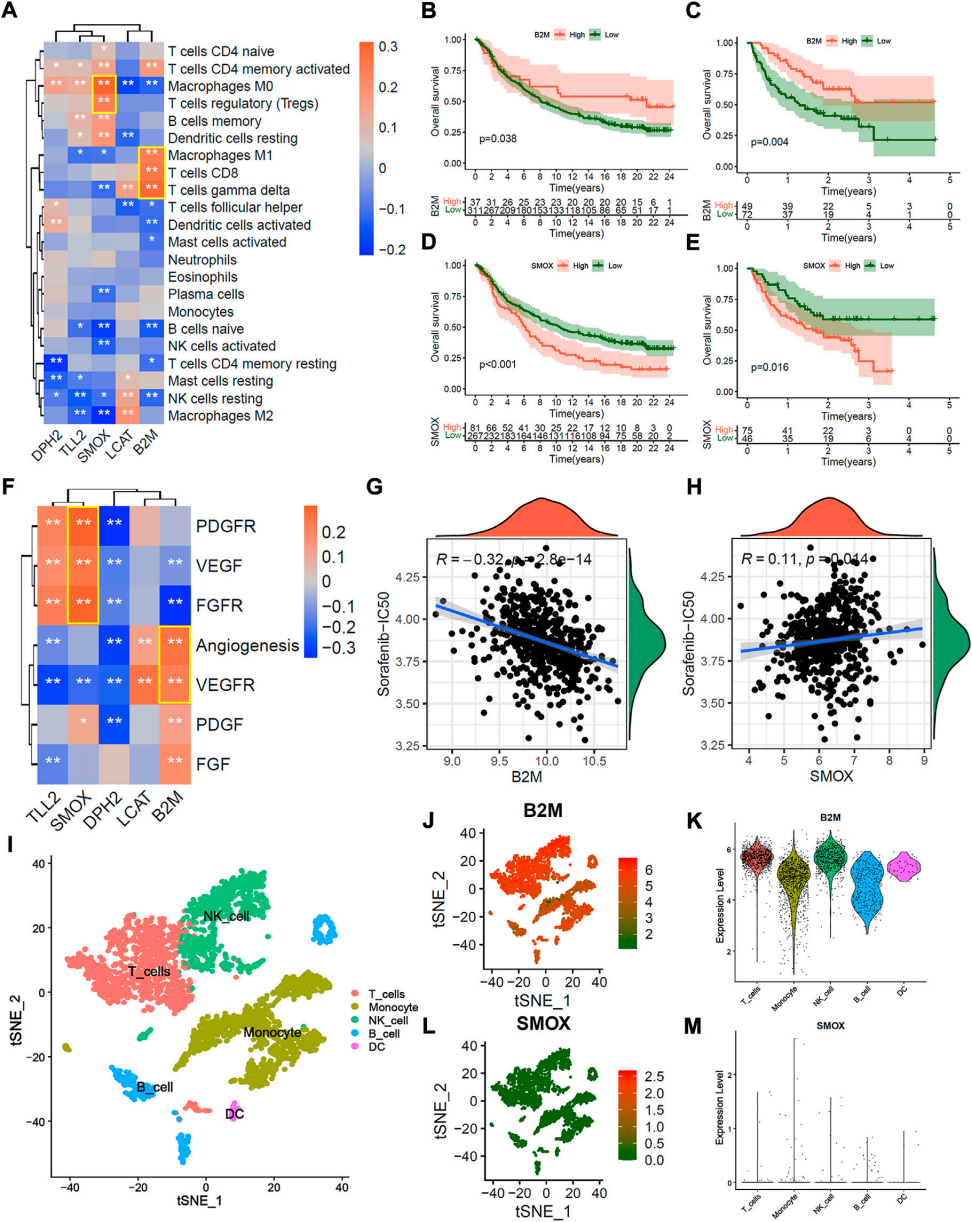
Potential mechanism of 5 hub genes in immunotherapy and anti-angiogenic therapy. **(A)** correlation heatmap between 5 hub genes and 22 infiltration immune cells. **(B**,**C)** Overall survival Kaplan-Meier analysis between patients with high and low B2M expression in IMvigor210 cohort **(B)** and Liu et al. Cohort **(C)**. **(D**,**E)** Overall survival Kaplan-Meier analysis between patients with high and low SMOX expression in IMvigor210 cohort **(D)** and Liu et al. Cohort **(E)**. **(F)** correlation heatmap of between 5 hub genes and the known vascular gene signatures score. **(G**,**H)** Correlation scatter plot of Sorafenib-IC_50_ and the expression of B2M **(G)** and SMOX **(H)**. **(I)** The t-SNE plot of immune cell clusters in GSE140228 single immune cell cohort. **(J**,**K)** B2M expression in the t-SNE plot **(J)** and violin plot **(K)**. **(L**,**M)** SMOX expression in t-SNE plot **(L)** and violin plot **(M)**.

To explore the potential mechanism of 5 hub genes in immunotherapeutic and anti-angiogenic therapy. We first explored the correlation between 5 hub genes and immune cell infiltration. The correlation heatmap showed that the expression of B2M is significantly positively correlated with activate immune cells, such as M1 macrophages, CD8^+^ T cells, and gamma delta (γδ) T cells. And the expression of SMOX is significantly positively correlated with suppressive immune cells (Figure 8A, *p* < 0.01, Spearman correlation test). As expected, the patients in IMvigor210 and Liu et al. cohorts with high B2M expression presented significant prolong overall survival probability (Figures 8B,C; all *p* < 0.05, Log-rank test), and the patients with high SMOX led to poor immunotherapeutic benefit (Figures 8D,E; all *p* < 0.05, Log-rank test). In addition, the expression of B2M is significantly positively correlated with angiogenesis and VEGFR signatures, while SMOX is significantly positively correlated with PDGFR, VEGFR and FGFR signatures (Figure 8F, *p* < 0.01, Spearman correlation test). As expect, the expression of B2M were significantly positively correlated with Sorafenib-IC_50_ (Figure 8G, R = −0.32, *p* < 0.01, Spearman correlation test), and the expression of SMOX were significantly negatively correlated with Sorafenib-IC_50_ (Figure 8G, R = 0.11, *p* < 0.01, Spearman correlation test).

We further explored the distribution of 5 hub genes in single immune cells from tumor tissue, peritumoral normal liver tissue, blood, and ascites of HCC patients. Figure 8I presented the t-SNE plot of immune cell clusters in the GSE140228 single immune cell cohort. The t-SNE plot and violin plot showed that B2M was over-expressed in most immune cells (Figures 8J,K), while the SMOX was down-expressed (Figures 8L,M). Similar results were found in tumor tissue, peritumoral normal liver tissue, blood and ascites, respectively (Supplementary Figures S9A–L). The patients in IMvigor210 and Liu et al. cohorts with high expression of DPH2 were associated with immunotherapeutic benefits (Supplementary Figures S10A,B; all *p* < 0.05, Log-rank test), but the expression of LCAT and TLL2 were not significantly associated with immunotherapeutic response (Supplementary Figures S10D–E,G,H; all *p* > 0.05, Log-rank test). Besides, the expression of DPH2 was not significantly correlated with Sorafenib-IC_50_ (Supplementary Figure S10C, *p* = 0.9, Spearman correlation test), and the expression of LCAT was significantly negatively correlated with Sorafenib-IC_50_ (Supplementary Figure S10F, R = −0.25, *p* < 0.001, Spearman correlation test), and the expression of TLL2 were significantly positively correlated with Sorafenib-IC_50_ (Supplementary Figure S10I, R = 0.15, *p* < 0.001, Spearman correlation test).

## Discussion

In the present study, we elaborated on the relevance in terms of m6A modification and the immune microenvironment of liver cancer. First, we identified three distinct m6Aclusters with different tumor microenvironment. The m6Acluster-A was classified as an immune-inflamed phenotype with lymphocyte infiltration, which may predict a better immune response (Galon and Bruni, 2019). The m6Acluster-B was classified as an immune-desert phenotype, and m6Acluster-C was classified as an immune-excluded phenotype with stromal activation. We subsequently constructed a 5 genes prognosis signature termed as “m6Asig-Score” to evaluate the overall survival risk of individual HCC patients. As expected, the m6Asig-Score in immune-excluded and immune-desert phenotypes was high, as opposed to immune-inflamed phenotype. Further analyses illustrated that the high m6Asig-Score was associated with poor prognosis and advanced TNM stage, suggesting that the m6Asig-Score may serve as a new potential prognostic marker. Of note, the alteration of TP53 at a higher frequency and poorer immune response was also detectable in the high m6Asig-Score group, consistent with the previous study that TP53 gene mutation could down-regulated HCC immune response (Long et al., 2019). Besides, we discovered a clear correlation between the m6Asig-Score and immunotherapy response predictors such as IPS, TIS and TIDE (Charoentong et al., 2017; Danaher et al., 2018; Jiang et al., 2018), and validated it in two independent immunotherapy cohorts. Nowadays, anti-angiogenic drugs (VEGF inhibitors) combined with immune checkpoint inhibitors have become the first-line treatment against advanced HCC. Interestingly, the m6Asig-Score had a significant negative correlation with the VEGFR signature (details in method) as well as the sensitivity of anti-angiogenic drugs. And patients with H-VEGFR signature and L-m6Asig-Score presented the best survival benefit. This coincides with the combination strategies of immunotherapy and anti-angiogenic therapy in advanced HCC.

We further found that two of five m6Asig-Score inner genes, B2M and SMOX, were associated with immune cell infiltration, immune response, and sensitivity to sorafenib. B2M, as a crucial ingredient of MHC class I–mediated antigen presentation by tumor cells, has been announced to be presented in immune cells (Pereira et al., 2017; Wang et al., 2021). It is noteworthy that the alteration of B2M gene could prevail in the emergence of T-cell-based immunotherapy resistance (Riaz et al., 2017; Hellmann et al., 2018). On the contrary, the B2M with increased expression correlated with a more predominant immune response along with survival benefits (Martinez-Morilla et al., 2021). In our study, the patients with high B2M expression were enriched in CD8^+^ T cell infiltration and associated with better sorafenib sensitivity, which prompted better immune and anti-angiogenic efficacy. Similarly, our study also showed that the patients with high B2M expression were dominated by CD8^+^ T cell infiltration and exhibited higher sorafenib sensitivity, which prompted better immune and anti-angiogenic efficacy. SMOX is generally a critical polyamine catabolic enzyme, by which the polyamine spermine can be metabolized into spermidine plus H2O2, giving rise to inflammation and carcinogenesis (Gobert et al., 2018; Sierra et al., 2020). Several studies denoted that the SMOX was overexpressed and accelerated tumor growth in HCC or NSCLC patients (Hu et al., 2018; Huang et al., 2021). In our study, the patients with high SMOX were enriched in regulatory T cell (Tregs) infiltration and associated with disappointing sorafenib sensitivity, which prompted poor immune and anti-angiogenic efficacy.

Although we systematically reviewed the literature and selected 26 m6A methylation regulators, more new finding regulators should be incorporated to enhance our understanding of m6A methylation modification. The HCC cohorts in our study were collected from public datasets, which lacked clinical and sub-clinical information on sorafenib efficacy. In addition, in the absence of appropriate HCC immunotherapy datasets, we hope that two metastatic urothelial carcinoma and melanoma immunotherapy cohorts could verify the prediction of m6Asig-Score, and strengthen our findings. The m6Asig-Score was significantly negatively correlated with anti-angiogenic drug sensitivity.

## Conclusion

All in all, our study indicated that the m6Asig-Score, B2M, and SMOX may act as new potential biomarkers in predicting immunotherapeutic and anti-angiogenic therapy responses. There are still needed more prospective HCC cohorts under immune and anti-angiogenesis drugs treatment to validate our conclusions. And more clinicopathological characteristics should be considered to improve the prediction accuracy.

## Data Availability

The original contributions presented in the study are included in the article/Supplementary Material, further inquiries can be directed to the corresponding author.
