# Supplementary material for: Construction of m6A-based prognosis signature and prediction for immune and anti-angiogenic response
Source: Front Mol Biosci. 2022 Oct 21;9:1034928. doi: 10.3389/fmolb.2022.1034928 (PMC9634552; doi:10.3389/fmolb.2022.1034928)
Supplement: Supplementary file 1 [file Presentation1.PDF]

Supplement information

Figure S1. Prognostic analysis of 26 m6A regulators in in the TCGA-LIHC cohort. (A-B) Univariate Cox forest-plot of DFS (A) and OS (B) for 26 m6A regulators in the TCGA-LIHC cohort. Risk factors were depicted in Hazard ratio(log10) > 0 and protective factors were depicted in Hazard ratio(log10) < 0. DFS, disease free survival; OS, overall survival.

Figure S2. Unsupervised clustering of 24 m6A regulators. (A-E) Consensus matrices of the merged HCC cohort for k = 2-6. (F) PCA analyses for the three m6A modification patterns depicted by the dot in different colors. m6Aculster A, aquamarine, m6Aculster B, yellow; m6Aculster C, pink.

Figure S3. The expression of 24 m6A regulators and relevant biological pathway among three m6A modification patterns in HCC. (A) Comparison of expression in 24 m6A regulators among three distinct m6Aclusters. The upper and lower ends of the boxes represented interquartile range of values. The gene expression differences were compared via one-way ANOVA test. (B, C) Heatmap indicate the GSVA score based on KEGG pathways curated from MSigDB in m6Acluster B vs m6Acluster C (B) and m6Acluster B vs m6Acluster C (C).

Figure S4. Kaplan-Meier analysis of 5 hub genes in TCGA-LIHC training, TCGALIHC testing and GSE76427 cohorts. (A-O) Kaplan-Meier analysis of overall survival (OS) for B2M, LCAT, DPH2, SMOX and TLL2 respectively in TCGA-LIHC training cohort, testing cohort and GSE76427 cohort (Log-rank test). (A-C) B2M, (DF) LCAT, (G-I) DPH2, (J-L) SMOX, (M-O) TLL2.

Figure S5. Overall survival Kaplan-Meier curves for high and low m6Asig-Score patient groups in different age and TNM stage subgroups. (A-D) Kaplan-Meier analysis of overall survival for high and low m6Asig-Score in age ≤ 60 (A), age > 60 (B), TNM stage I/II (C), and TNM stage III/IV (D) subgroups (Log-rank test). Red line presented high m6Asig-Score, while light green line presented low m6Asig-Score.

Figure S6. Immune and angiogenesis microenvironment characteristics in two m6Asig-Score subgroups. (A) The fraction of TIME cells infiltration in two m6AsigScore subgroups (Wilcoxon test). (B) The vascular genes expression in two

m6Asig-Score subgroups (Student' t-test). (C) Spearman correlations analysis between m6AsigScore and TIME infiltrating cells signatures in merged HCC cohort. The dot in red presented positive correlation, and the dot in green presented negative correlation. The size of the dot indicated the strength of the correlation. (D) Spearman correlations analysis between m6Asig-Score and the 21 vascular-related genes in merged HCC cohort using Spearman analysis. green: negative correlation; red: positive correlation.

Figure S7. correlation analysis of m6Asig-Score and 5 hub gene with Pazopanib-IC50 and the target genes of sorafenib. (A) Spearman correlation analysis of m6Asig-Score and Pazopanib-IC50. (B) The expression correlation heatmap between 5 hub genes and the known Sorafenib target genes. The block in red means positive correlation, while the block in blue means negative correlation. *, P<0.05; *, P<0.01.

Figure S8. Scatters correlation plot between the m6Asig-Score and the known vascular gene signatures. (A) Spearman correlations analysis between the known vascular related gene signatures and m6Asig-Score in merged HCC cohort. The dot in red presented positive correlation, and the dot in green presented negative correlation. The size of the dot indicated the strength of the correlation. (B-H) Spearman correlation analysis of Sorafenib-IC50 and VEGFR, FGR, PDGFR, VEGF, FGFR, PDGF and Angiogenesis. (B) VEGF, (C)FGF, (D) FGFR, (E) PDGF and (F) PDGFR, (G) Angiogenesis

Figure S9. Single immune cells landscape of B2M and SMOX in HCC. (A-C) The t-SNE plot of immune cell landscape (A), B2M (B) and SMOX (C) expression of in GSE140228 normal cohort. (D-F) The t-SNE plot of immune cell landscape (D), B2M (E) and SMOX (F) expression of in GSE140228 tumor cohort. (G-I) The t-SNE plot of immune cell landscape (G), B2M (H) and SMOX (I) expression of in GSE140228 tumor cohort. (J-L) The t-SNE plot of immune cell landscape (J), B2M (K) and SMOX (L) expression of in GSE140228 tumor cohort.

Figure S10. Prediction of DPH2, LCAT and TLL2 in immunotherapy and antiangiogenic therapy. Overall survival Kaplan-Meier analysis of DPH2, LCAT and TLL2 in IMvigor210 cohort (A, D, G) and Liu et al. Cohort (B, E, H). Correlation scatter plot of Sorafenib-IC50 and the expression of DPH2, LCAT and TLL2 (C, F, I).

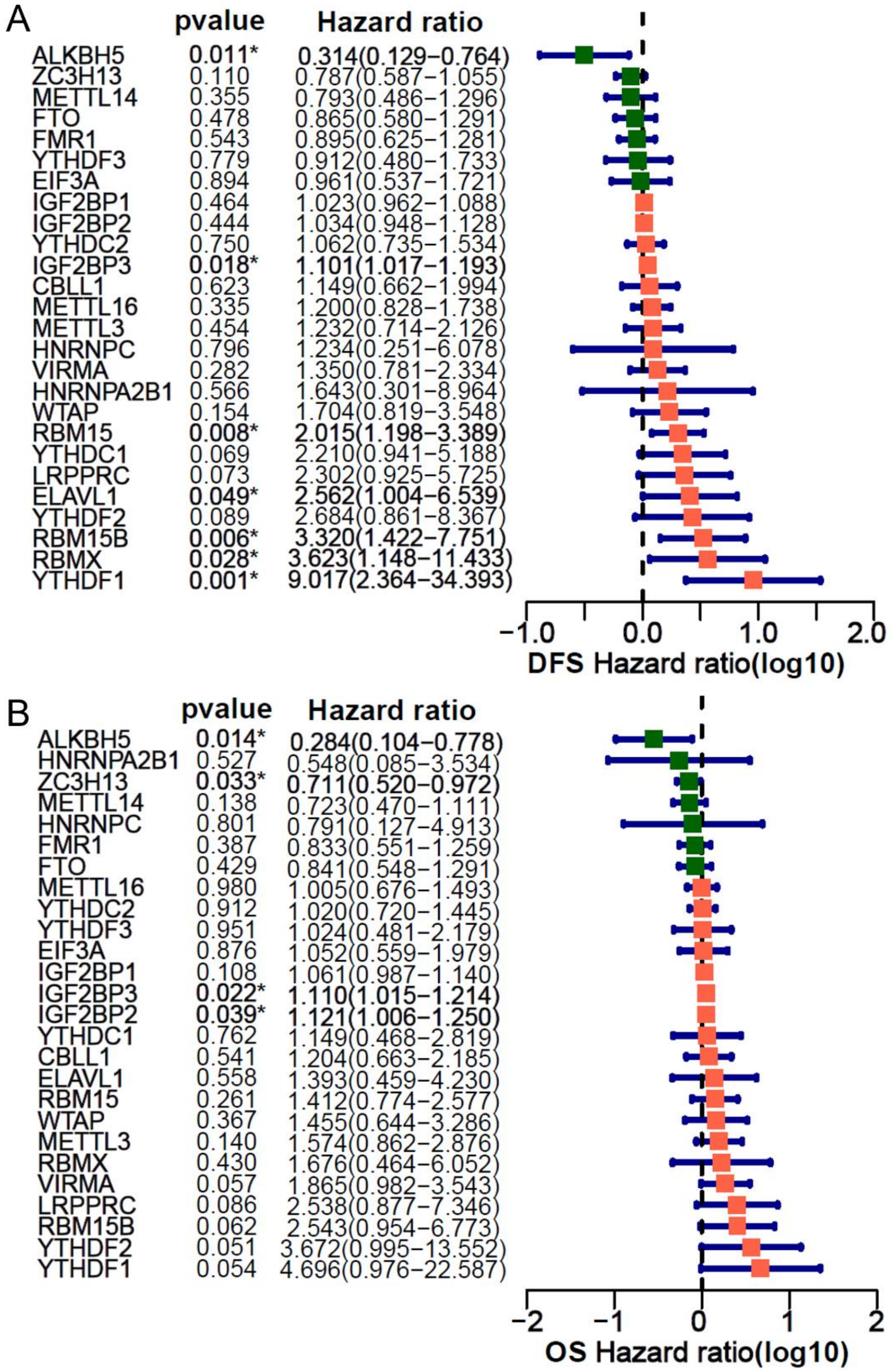

Figure S1. Prognostic analysis of 26 m6A regulators in in the TCGA-LIHC cohort.

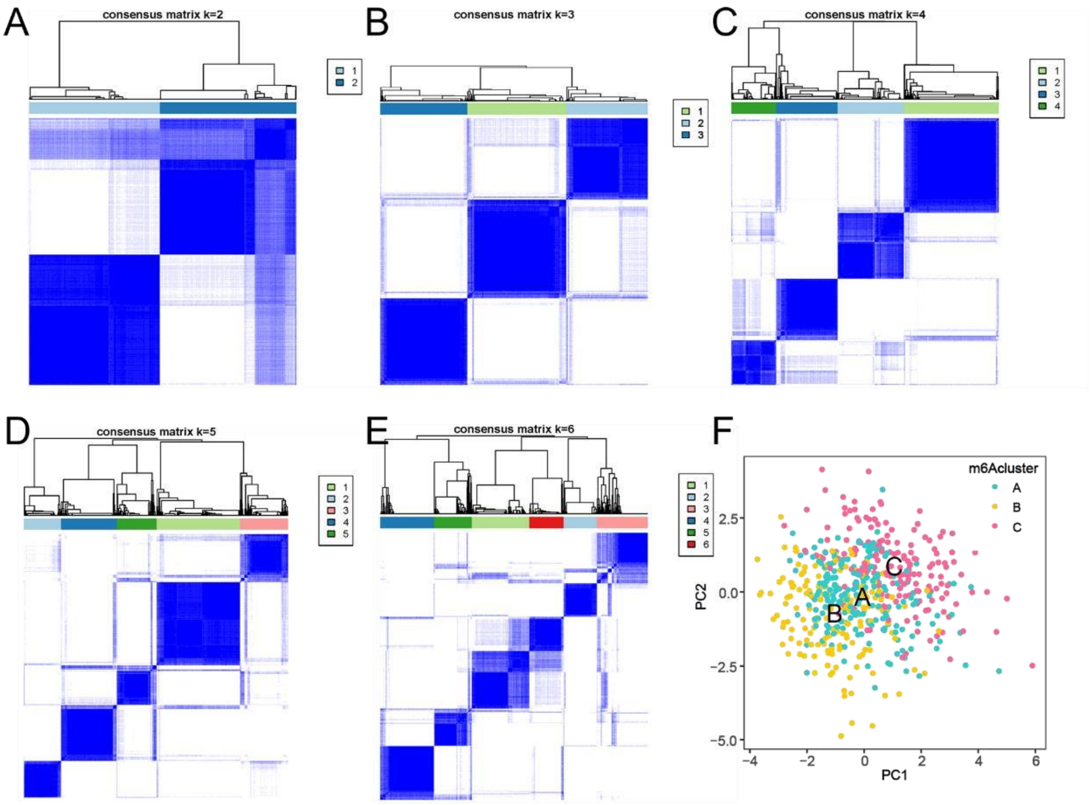

Figure S2. Unsupervised clustering of 24 m6A regulators.

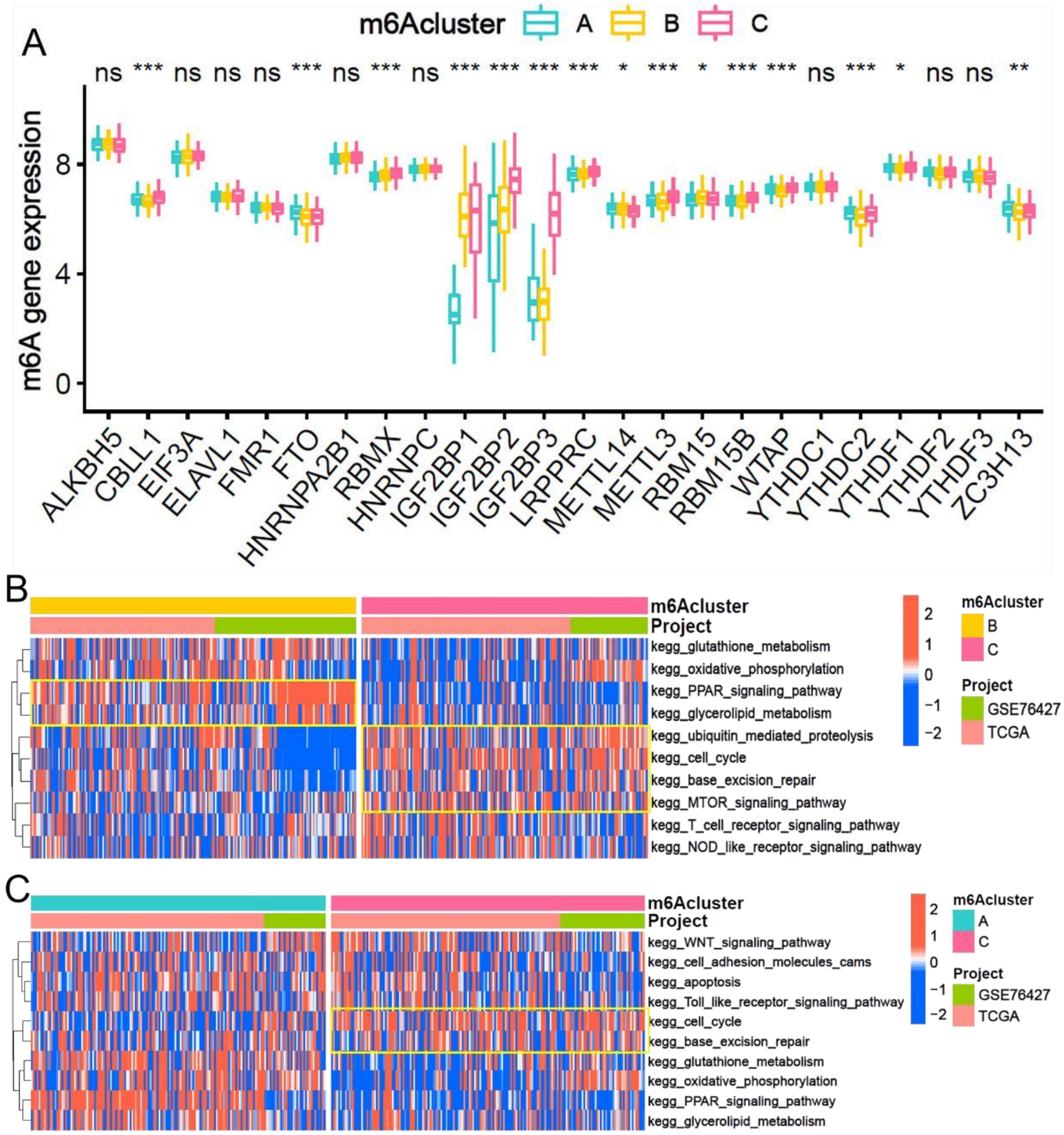

Figure S3. The expression of 24 m6A regulators and relevant biological pathway among three m6A modification patterns in HCC.

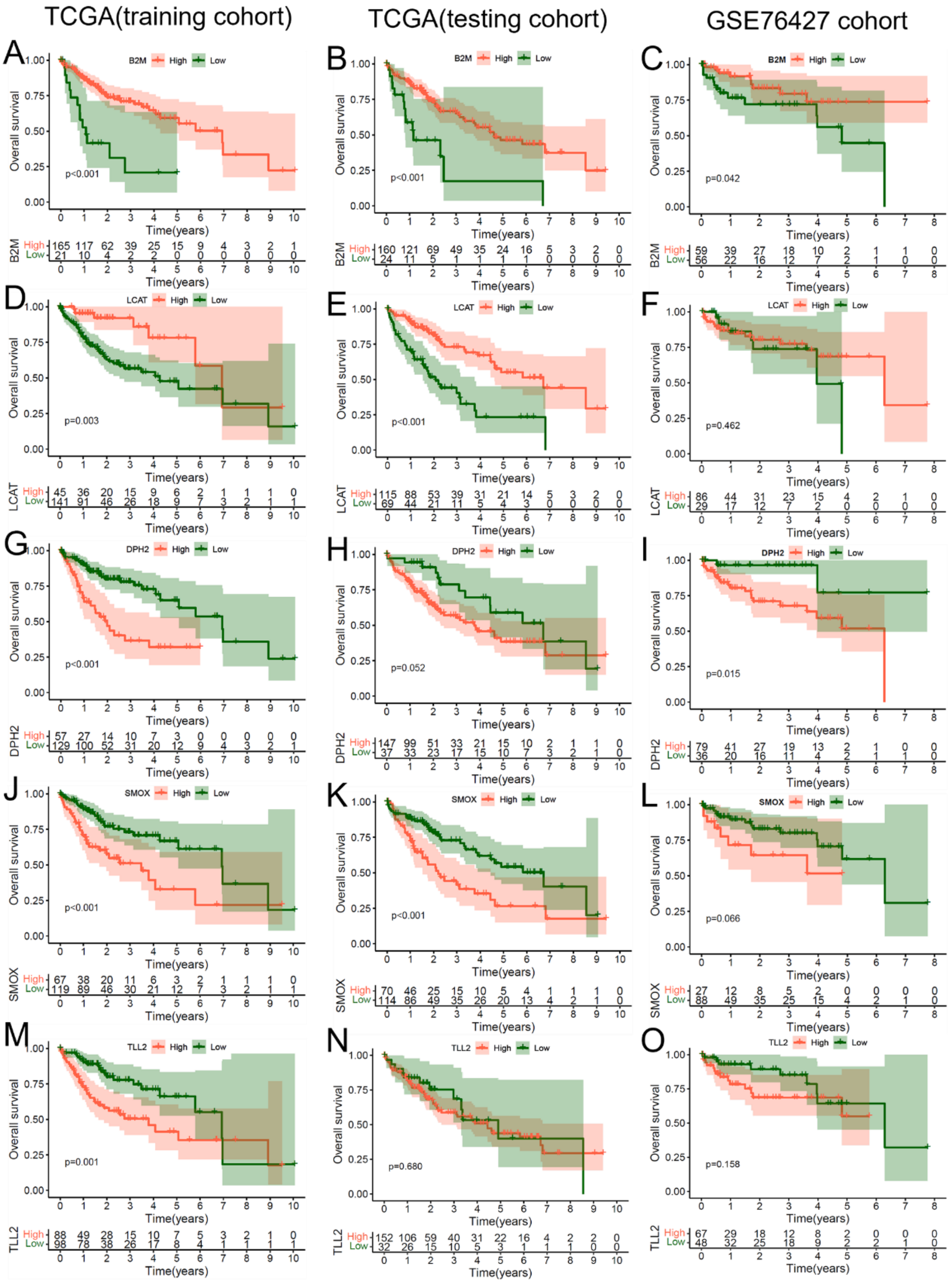

Figure S4. Kaplan-Meier analysis of 5 hub genes in TCGA-LIHC training, TCGA-LIHC testing and GSE76427 cohorts.

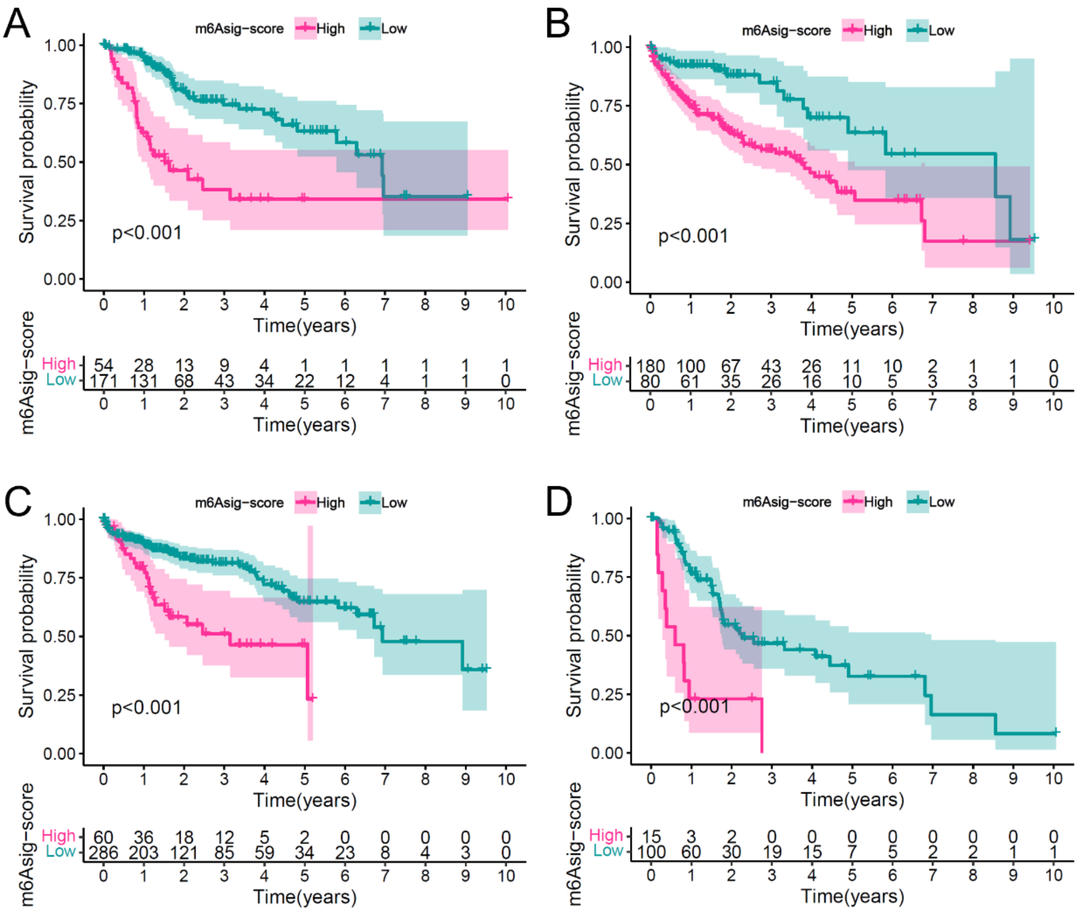

Figure S5. Overall survival Kaplan-Meier curves for high and low m6Asig-Score patient groups in different age and TNM stage subgroups.

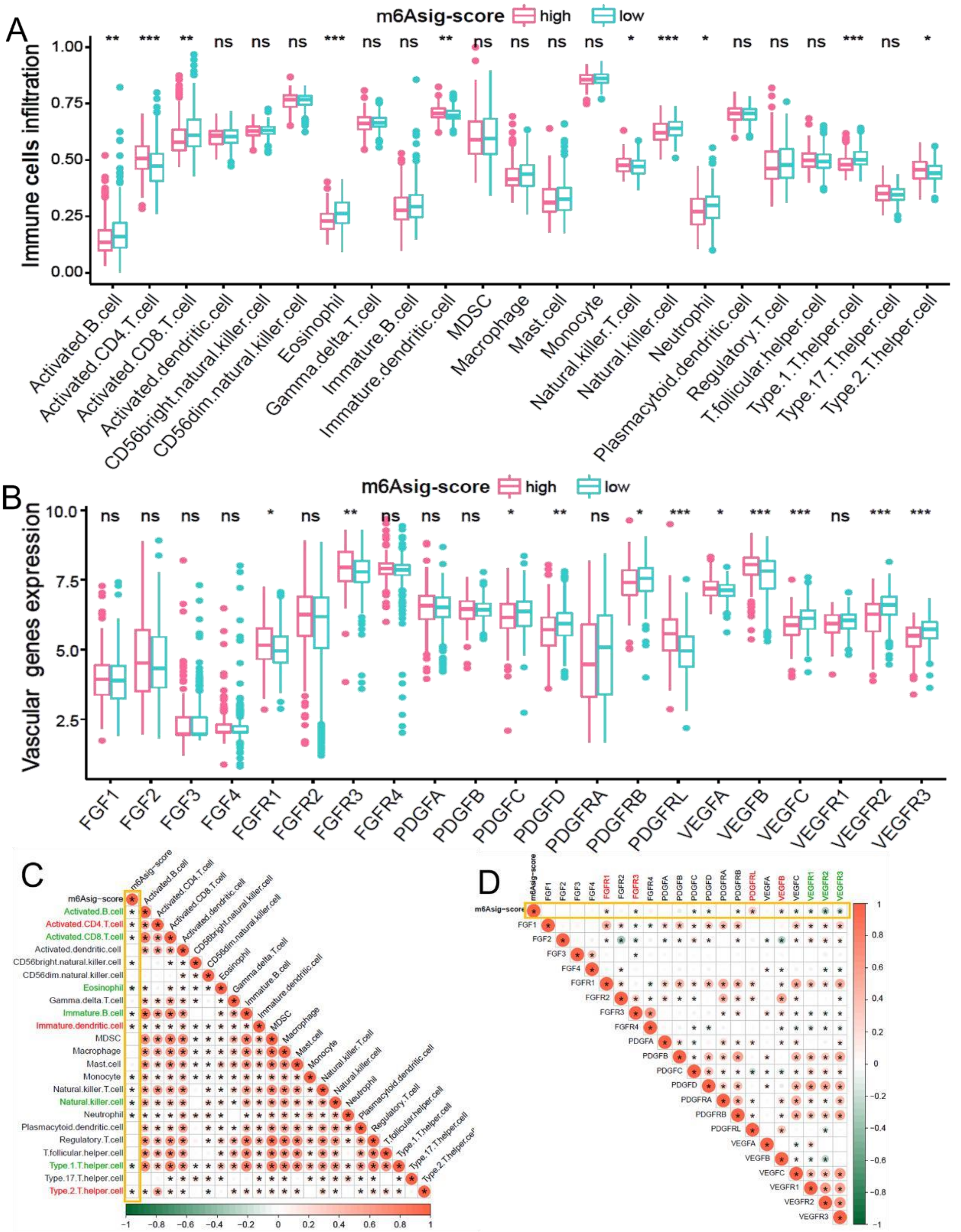

Figure S6. Immune and angiogenesis microenvironment characteristics in two m6Asig-Score subgroups.

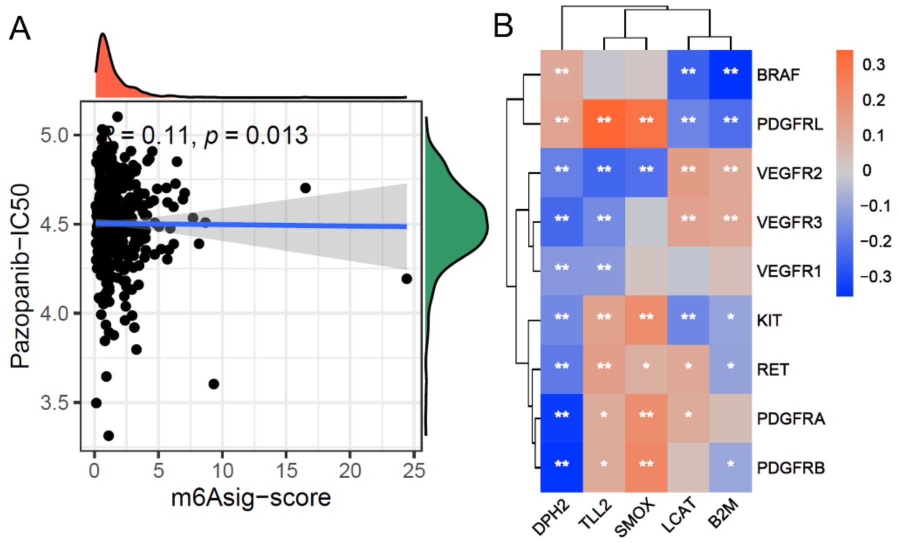

Figure S7. correlation analysis of m6Asig-Score and 5 hub gene with Pazopanib-IC50 and the target genes of sorafenib.

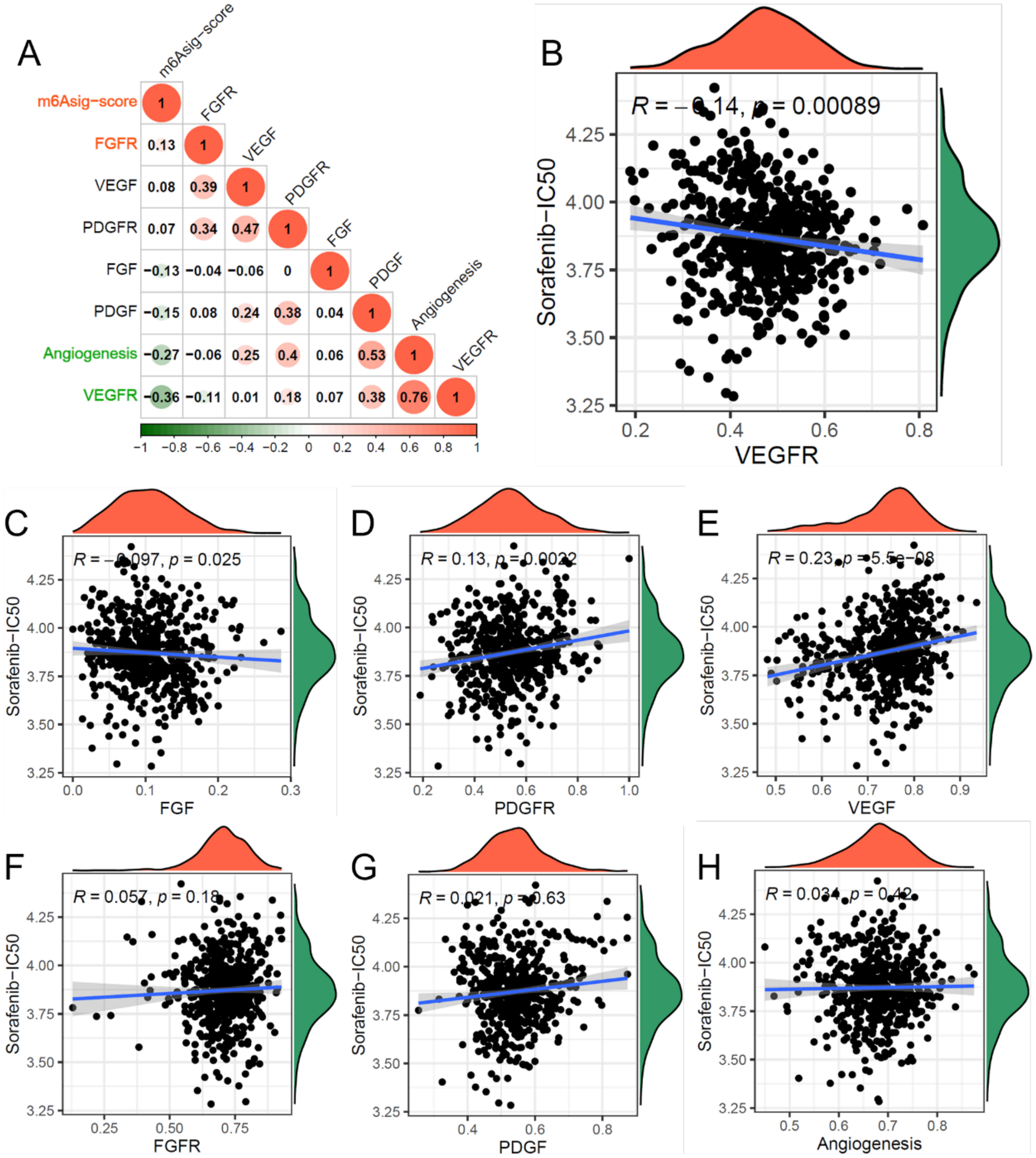

Figure S8. Scatters correlation plot between the m6Asig-Score and the known vascular gene signatures.

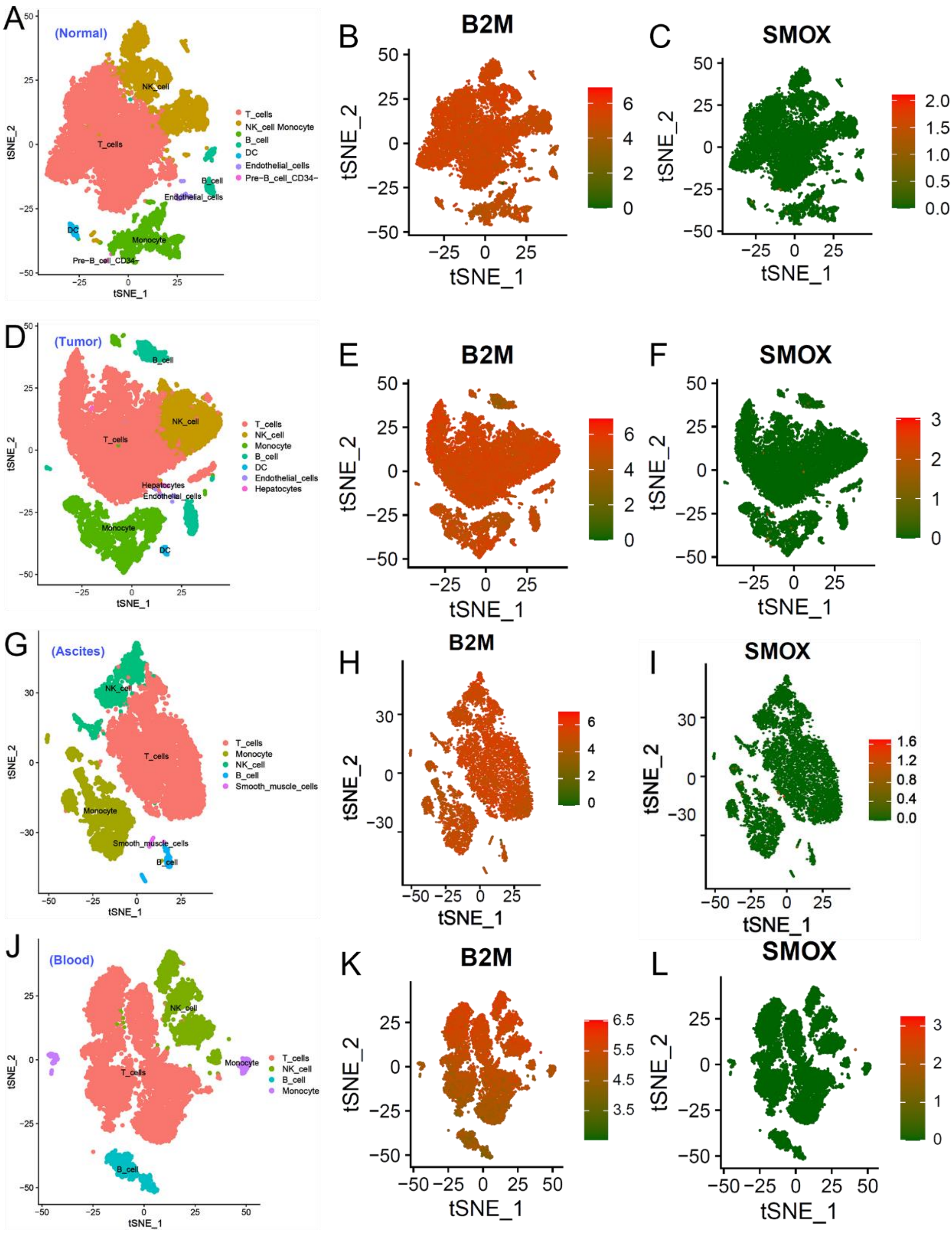

Figure S9. Single immune cells landscape of B2M and SMOX in HCC.

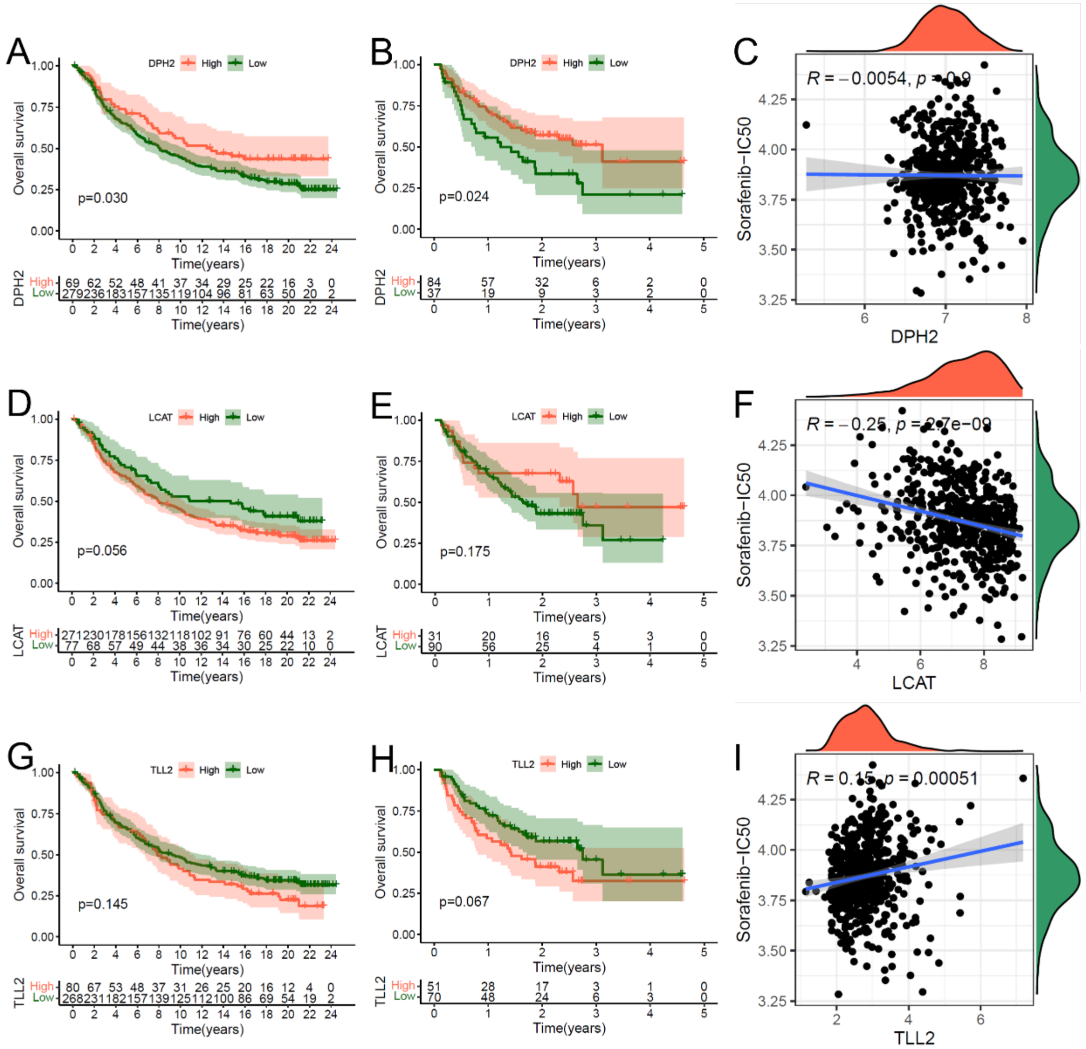

Figure S10. Prediction of DPH2, LCAT and TLL2 in immunotherapy and antiangiogenic therapy.
